# Inter-Species Grafting Caused Extensive and Heritable Alterations of DNA Methylation in *Solanaceae* Plants

**DOI:** 10.1371/journal.pone.0061995

**Published:** 2013-04-16

**Authors:** Rui Wu, Xiaoran Wang, Yan Lin, Yiqiao Ma, Gang Liu, Xiaoming Yu, Silin Zhong, Bao Liu

**Affiliations:** 1 Key Laboratory of Molecular Epigenetics of Ministry of Education (MOE), Northeast Normal University, Changchun, Jilin Province, China; 2 Jilin Academy of Vegetables and Flowers, Changchun Changchun, Jilin Province, China; 3 Department of Plant Biology, Carnegie Institution for Science, Stanford, California, United States of America; 4 Boyce Thompson Institute for Plant Research, Cornell University, Ithaca, New York, United States of America; Cankiri Karatekin University, Turkey

## Abstract

**Background:**

Grafting has been extensively used to enhance the performance of horticultural crops. Since Charles Darwin coined the term “graft hybrid” meaning that asexual combination of different plant species may generate products that are genetically distinct, highly discrepant opinions exist supporting or against the concept. Recent studies have documented that grafting enables exchanges of both RNA and DNA molecules between the grafting partners, thus providing a molecular basis for grafting-induced genetic variation. DNA methylation is known as prone to alterations as a result of perturbation of internal and external conditions. Given characteristics of grafting, it is interesting to test whether the process may cause an alteration of this epigenetic marker in the grafted organismal products.

**Methodology/Principal Findings:**

We analyzed relative global DNA methylation levels and locus-specific methylation patterns by the MSAP marker and locus-specific bisulfite-sequencing in the seed plants (wild-type controls), self- and hetero-grafted scions/rootstocks, selfed progenies of scions and their seed-plant controls, involving three *Solanaceae* species. We quantified expression of putative genes involved in establishing and/or maintaining DNA methylation by q-(RT)-PCR. We found that (1) hetero-grafting caused extensive alteration of DNA methylation patterns in a locus-specific manner, especially in scions, although relative methylation levels remain largely unaltered; (2) the altered methylation patterns in the hetero-grafting-derived scions could be inherited to sexual progenies with some sites showing further alterations or revisions; (3) hetero-grafting caused dynamic changes in steady-state transcript abundance of genes encoding for a set of enzymes functionally relevant to DNA methylation.

**Conclusions/Significance:**

Our results demonstrate that inter-species grafting in plants could produce extensive and heritable alterations in DNA methylation. We suggest that these readily altered, yet heritable, epigenetic modifications due to interspecies hetero-grafting may shed one facet of insight into the molecular underpinnings for the still contentious concept of graft hybrid.

## Introduction

Available records testify that grafting was already discovered and used in ancient China more than 2,000 years ago, and since then the practice has been widely employed by horticulturists all over the world [1], [2]. Even nowadays, grafting is still an indispensable means for boosting performance of horticultural plants including many important vegetables and woody fruit trees [3]. This method is generally conducted by grafting the shoot part of a plant (scion) onto a root part of another plant (rootstock) often with distinct genetic constitution (different species or genera). Studies have shown that rootstocks could significantly improve some properties of scions by “passing” to them their own features, such as altered phenotypes of certain organs, improved production/quality, and elevated resistance to pests, diseases and abiotic stresses [4]–[9].

Despite its long history and prominent practical utility, the underlying mechanism for graft-induced phenotypic and physiological changes remains to be fully understood. It is generally accepted that metabolic substances including those that may produce large biological effects, such as hormones, proteins and signal molecules, could be transferred from one grafting partner to the other [10]. But whether the grafting process may cause changes that are sexually *heritable* has been historically controversial and remains so nowadays. Thus, when Darwin put forward the concept of “graft hybrid” meaning that asexual combination of different plant species may generate products that contain features of both grafting partners, and hence, are genetically distinct [11], they were met with skepticism, and being perceived as either non-existence or simply a kind of chimeras [1]. Nevertheless, circumstantial evidence demonstrating heritable phenotypic alterations in diverse grafted plants was persistently and widely reported [12]–[16], which however did not gain full credential due to lack of concrete mechanistic underpinnings [1].

Discovery of the highly transmissible nature of RNA molecules (including mRNAs and noncoding small (s) RNAs) between adjacent cells and across the whole plant [17], presumably via the plant intercellular connections called plasmodesmata, has opened up a fresh possibility for understanding the phenomenon of graft hybrids. For example, Kim [18] demonstrated that long-distance movement of mutant mRNA from the rootstock into the wild-type scions had caused a conspicuous change in leaf morphology, suggesting the translocated RNAs were functional. Various non-coding small RNAs have been shown to move over long distances via phloem sap in grafting [19]–[21]. In this regard, it has been proposed that if the transferred mRNAs (e.g., from rootstock) represent full-length retrotransposons, then conceivably they could be reverse-transcribed to produce cDNAs and be re-integrated into the new host (scion) genome, thus generating *bona fide* genetic changes [1]. So far, no empirical evidence is available to support this possibility, although it is theoretically viable. In addition to RNAs, recent studies have unequivocally demonstrated that plant grafting could indeed result in exchange of DNAs. For example, Stegemann and Bock showed that the plastid-encoded green fluorescent protein (GFP) marker was transferred from one grafting tobacco plant to another at the grafting junction [22]. Thyssen and colleagues have documented that interspecific somatic cell transfer of the entire 161-kb plastid genome was accomplished via grafting after clonal selection of the graft-junction somatic cells [23]. Similarly, complete plastid genome transfer between sexually incompatible plants was observed, as well as experimentally demonstrated, in natural grafts [24]. It should be noted however that in all the above cases, the transfer of plastid genomes appeared to be confined to the immediate graft junctions, which actually led Rusk to comment that these findings represented molecular evidence against the original meaning of graft hybrid [25].

DNA methylation is known to be sensitive and responsive to certain perturbations of internal and external conditions [26]. Given characteristics of the grafting process that entails intimate physical contact and imaginably myriad of chemical interactions between genetically divergent cells (hetero-grafting), the cellular environment is likely to be profoundly “perturbed” relative to that of the non-grafted controls. Conceivably, various epigenetic markers including DNA methylation, which are intrinsically metastable, might be induced to alter under the hetero-graft conditions [10]. This possibility gains an added weight given that long-distance transfer or interchange of RNA molecules between the grafting partners have been repeatedly documented to occur, discussed above. In addition, a specific kind of non-coding small RNAs, i.e., the 24 nt siRNAs are known to cause DNA methylation changes in a homology-dependent manner via the plant-specific RNA-directed DNA methylation (RdDM) pathway [26]–[28]. Indeed, a recent study has demonstrated in *Arabidopsis* that, a transgene-derived siRNAs as well as a substantial proportion of the endogenous siRNAs had moved across the graft union via plasmodesmata and phloem, and that the 24-nt mobile small RNAs directed DNA methylation in the genome of the recipient cells [29]. Similar results were also obtained in another study, also in *Arabidopsis* [30]. Nonetheless, how general and to what extent grafting may cause *de novo* DNA methylation alterations of endogenous genes, and whether the epigenetic alterations induced are heritable to organismal generations remained unknown.

We hypothesized that the grafting process between genetically divergent cells (hetero-grafting) might generate novel epigenetic marks in the scion, and a portion of which might be inherited to the next generation resulting in the the often-observed graft-hybrid phenotypes. To test this, we used methylation-sensitive amplified polymorphism (MSAP) markers to screen DNA methylation variations in the interspecific grafts involving three compatible species of the *Solanaceae* family (tomato, eggplant and pepper) that are widely used for grafting in horticultural production. We also used bisulfite Sanger sequencing to examine the heritability of the methylation status of a set of MSAP loci in the graft plants and their progenies.

## Results

### Relative global DNA methylation levels in both scions and rootstocks remained largely unaltered based on MSAP analysis

**Figure 1:**
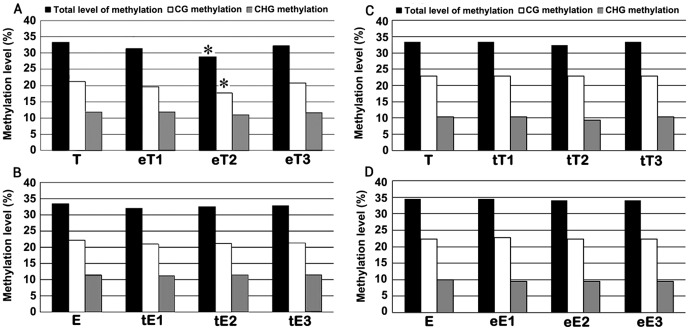
Tabulated data of relative DNA methylation levels based on the MSAP analysis. Relative levels of total, CG and CHG methylation at randomly sampled 5′-CCGG sites by the MSAP marker, in three independent tomato scions hetero-grafted to eggplant (marked as eT1-3) (**A**), three independent eggplant scions hetero-grafted to tomato (marked as tE1-3) (**B**), three independent self-grafted tomato scions (marked as tT1-3) (**cC**), three independent self-grafted eggplant scions (marked as eE1-3) (**D**),and their respective seed-plant controls of tomato (T) and eggplant (E), respectively. * Values significantly lower than the values of the corresponding seed-plant controls (*P*<0.05).

Using 25 pairs of selective *EcoR*I + *Hpa*II/*Msp*I primer combinations (Table S1), 843 and 764 clear and reproducible (between two technical replicates, see *Methods*) bands were amplified across the set of plant samples (including control seed-plants and three individual scion plants) (Table S2). By tabulating the number of bands representing the various types of MSAP patterns, the relative CG, CHG and total (adding up the two) methylation levels were calculated (Figure 1 and Table S2). Compared with the non-grafted seed-plant control, all three kinds of methylation levels in the hetero-, reciprocally grafted scions (between tomato and eggplant) remained largely unchanged except in one (eT2) of the three tomato scions, in which both CG and total methylation levels were significantly reduced (Figure 1A and B; U_total_ = 2.26, >1.96; U_CG_ = 2.25, >1.96). Similarly, we did not detect large difference in methylation levels between the pepper rootstocks and their seed-plant controls (Figure S1). Expectedly, no alteration in methylation level was detected in three scions of self-grafted tomato (tT1-3) and eggplant (eE1-3), respectively (Figure 1C and D). This suggests that grafting (both self and hetero) did not cause a general alteration in DNA methylation levels in *Solanaceae* plants, although in specific cases (e.g., in scion eT2, Figure 1A) grafting may cause a significant global reduction in DNA methylation levels.

### Altered local DNA methylation patterns in grafted scions and rootstocks detected by MSAP analysis

**Figure 2:**
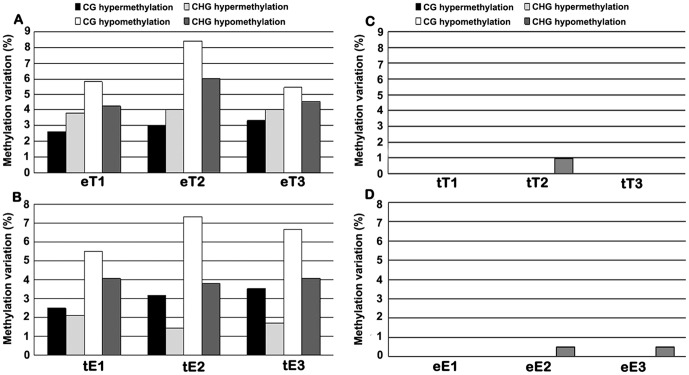
Tabulated data of alteration in DNA methylation patterns based on the MSAP analysis. Alterations in the four major methylation patterns, CG hyper, CHG hyper, CG hypo and CHG hypo, in three independent tomato scions hetero-grafted to eggplant (marked as eT) (**A**), three independent eggplant scions grafted to tomato (marked as tE) (**bB**), three independent self-grafted tomato scions (**C**), and three independent self-grafted eggplant scions (**D**) was calculated based on the MSAP data by comparing with their respective seed-plant controls of tomato and eggplant, respectively.

The relative global DNA methylation levels, described above, represented *collective* values of all the 5′-CCGG sites sampled by the MSAP marker. Therefore, methylation alterations in opposite directions, i.e., hypo and hyper, might be offsetting each other and be reflected as an insignificant net change in methylation levels. Hence, we have examined the MSAP profiles in a locus-specific manner, namely scoring the loss of original bands and gain of novel bands in the scion plants relative to their seed-plant controls. Because the cultivars representing the three species of *Solanaceae* are pure lines (Materials and methods), we used one seed-plant of each cultivar as non-grafted controls, and lack of inter-plant polymorphism for methylation patterns were further verified by testing 15 randomly chosen plants for each cultivar (data not shown). We found alterations at frequencies ranging from 1.4% to 8.4% of the four major methylation patterns that can be scored by MSAP [31], i.e., CG-hyper, CHG-hyper, CG-hypo and CHG-hypo, occurred in the hetero-grafts eT1-3 (tomato grated to eggplant rootstock) and tE1-3 (eggplant grated to tomato rootstock) (Figure 2A and B). We noted that among the four patterns, CG hypomethylation occurred at the highest frequencies in all the scions studied (ranging from 5.5% to 8.4%). It is consistent with our observation that the eT2 scions have lower relative global methylation level was, as described above (Figure 1). In addition, the overall hypo- and hypermethylation alterations are indeed largely offsetting each other and rendering the collective net methylation levels virtually unchanged in the scions (except for eT2) (Figure 1). Despite our study primarily focused on the scion section that is of significant agricultural values, we also observed similar DNA methylation alterations in the examined pepper rootstocks (with tomato as the scions) (Figure S1). In contrast to these hetero-grafts, none or very infrequent alteration in DNA methylation patterns was detected in the self-grafts of tomato (tT1-3) and eggplant (eE1-3) (Figure 2C and D).

To explore possible functional relevance of the loci with DNA methylation alterations, we isolated and sequenced a subset of variant MSAP bands from scions or rootstocks. Based on a BlastX analysis, we found that of the 54 variant MSAP bands that gave quality sequencing, 51.9% showed significant homology to protein-coding genes with known functions (Table 1, Table S4). Intriguingly, we have identified sequences corresponding to two disease resistance-related proteins (RP3 and ST27), while grafting were often used for enhance disease resistance traits in horticulture practices.

**Table 1:** Functional classification of cloned MSAP bands showing alterations in DNA methylation pattern in grafted plants based on BlastX.

Classification	Number of variant bands	Percentage of variant bands
Known-function gene	28	51.9%
Putative protein-coding gene	12	22.2%
Transposon/retrotransposon	4	7.4%
No similarity	10	18.5%
Total	54	100%

### Some DNA methylation alterations in the grafted scions are heritable to their self-pollinated progenies

To test whether the altered DNA methylation patterns are heritable, we monitored the transmission of 15 variant MSAP loci that occurred in the tomato scions or eggplant scions to their respective self-pollinated progenies. Ten selfed progenies for each grafted scion plant were examined using the same MSAP primer pairs, and frequencies of inheritance (the number of progenies showing inheritance of the modified methylation patterns as detected in the scion mother plant), reversion (the number of progenies showing methylation patterns being reversed back to original patterns), and further alterations (the number of progenies showing further alterations), were tabulated and divided by the total number of progeny plants analyzed (Table 2). We found that eight of out the 15 loci showed complete inheritance (100% frequency) of the altered patterns, three showed high level of inheritance (frequencies ranging from 83.3% to 96.7%) of the altered patterns, and only four showed none or minimal (frequencies ranging from 0 to 16.7%) inheritance of the altered patterns, that is, the altered patterns being predominately reverted back to the control patterns in these four plants (Table 2). For two loci, further alteration of the already altered methylation patterns were noted, and both are to the same changing direction (Table 2).

**Table 2:** Altered DNA methylation patterns of 15 variant MSAP bands in the grafted tomato or eggplant scions, their predicted functional homology and their inheritance to selfed progenies.

Variant MSAP band	Scion	Pattern alteration in grafted plant	Behavior of variant bands in progenies			Functional homology based on BlastX
			Inherit.	Rev.	Further alter.	
ST1	Tomato	CG-hypo	93.3%	6.7%	0	No homology
ST3	Tomato	CHG-hypo	100%	0	0	Photosystem II CP47 chlorophyll apoprotein [*Terminalia catappa*] gb|ABV65673.1|
ST4	Tomato	CG-hyper	0	96.7%	3.3% (CHG- hyper)	Putative senescence-associated protein [*Pisum sativum*] dbj|BAB33421.1|
ST5	Tomato	CG-hypo	0	100%	0	Kelch repeat-containing F-box family protein [*Arabidopsis thaliana*] ref|NP_565238.1|
ST8	Tomato	CG/CHG-hyper	0	100%	0	No homology
ST9	Tomato	CHG-hypo	16.7%	83.3%	0	Ribosomal protein L16 [*Solanum lycopersicum*] ref|YP_514889.1|
ST10	Tomato	CG/CHG- hyper	100%	0	0	No homology
ST11	Tomato	CG-hyper	100%	0	0	No homology
ST13	Tomato	CHG-hypo	96.7%	3.3%	0	ABC transporter [*Vibrionales bacterium*SWAT-3]ref|ZP_01812900.1|
SE1	Eggplant	CHG-hyper	100%	0	0	Hypothetical protein SpolCp017 [*Spinacia oleracea*] ref|NP_054927.1|
SE2	Eggplant	CHG-hypo	100%	0	0	Putative retrotransposon protein, [*Solanum demissum*] gb|AAT38724.1|
SE3	Eggplant	CG/CHG-hypo	100%	0	0	Cation-chloride co-transporter [*Nicotiana tabacum*] gb|AAC49874.1|
SE4	Eggplant	CG-hypo	83.3%	0	16.7% (CHG- hypo)	No homology
SE5	Eggplant	CHG-hyper	100%	0	0	No homology
SE16	Eggplant	CG-hyper	100%	0	0	Hypothetical protein Mp023 [*Nicotiana tabacum*] ref|YP_173370.1|

### Validation of graft-induced DNA methylation alterations and their heritability by bisulfite Sanger-sequencing

The alteration in DNA methylation patterns revealed by the MSAP assay is limited to the 5′-CCGG sites. In addition, the MSAP assay is liable to amplification artifacts, although in our case this should be minimized given that two independent technical replicates were used and only completely reproducible bands were scored (see Methods).To validate the MSAP-detected methylation pattern alterations and their heritability, as well as to explore whether the adjacent cytosine residues might be affected by grafting, we performed bisulfite Sanger-sequencing on three of the variant MSAP bands, ST4, SE1 and SE2, which were isolated from tomato or eggplant scions (Table 2).

**Figure 3:**
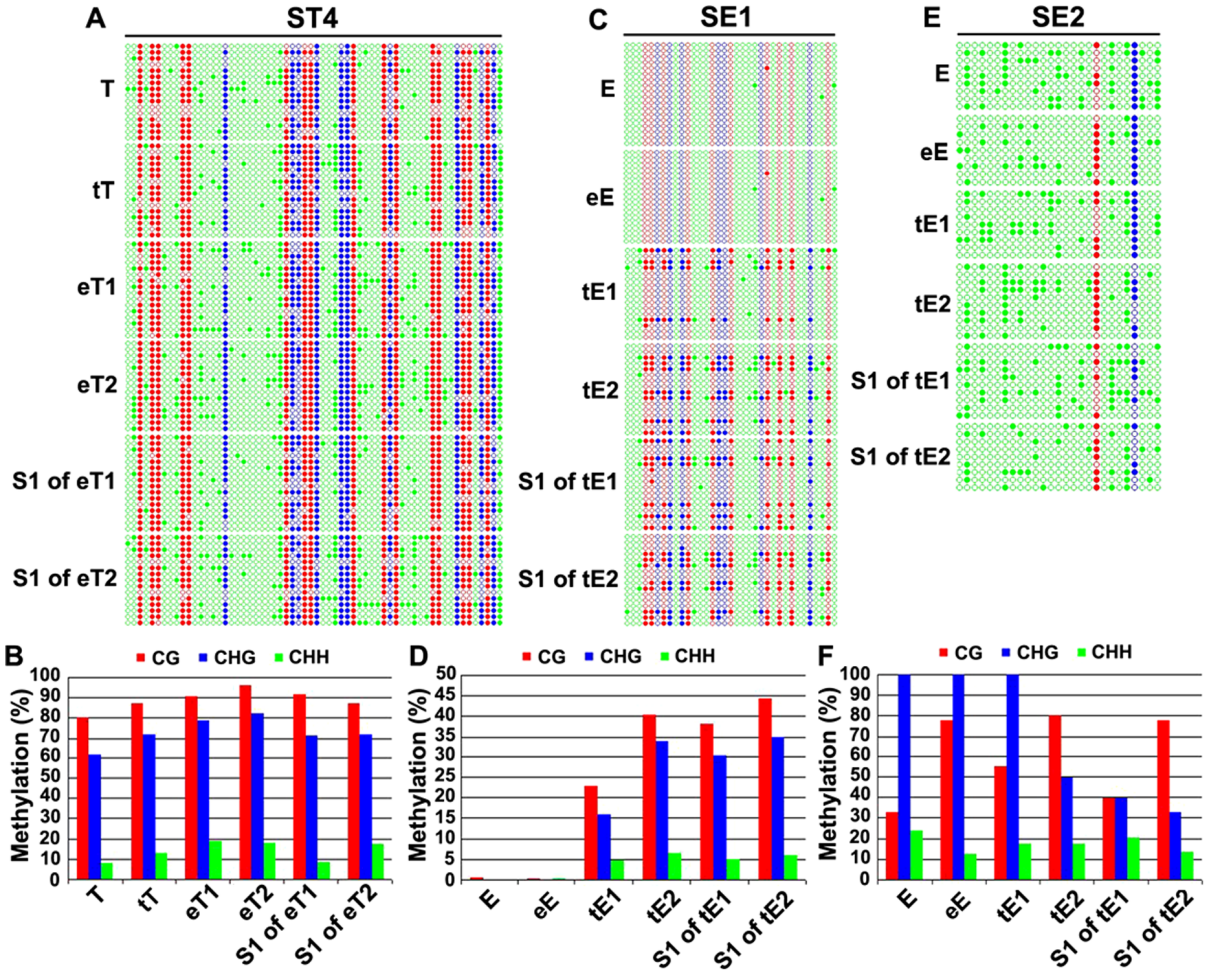
Validation of grafting-induced DNA methylation alterations and their inheritance by locus-specific bisulfite Sanger-sequencing. DNA methylation maps and collective methylation values (in percentage) for the three selected MSAP bands, unique-copy genic sequences ST4 (from tomato scion) (A and B) and SE1 (from eggplant scion) (C and D), and a low-copy retrotransposon fragment SE2 (from eggplant scion) (E and F), determined by genomic bisulfite Sanger-sequencing in the following samples: seed-plants of tomato (seed-plant T) and eggplant (seed-plant E), two independent, reciprocally hetero-grafted scion plants, tomato grafted to eggplant (eT1-2) and eggplant grafted to tomato (tE1-2), and one selfed progeny (S1) for each scion-plant of tomato (tT) and Eggplant (eE). All three types of cytosines, CG (red circles), CHG (blue circles) and CHH (green circles) were shown in the map. Filled and empty circles indicate methylated and unmethylated cytosines, respectively. The red, blue and green columns in the histograms refer to the collective methylation levels (in percentage) respectively of CG, CHG, and CHH for each sample, described above.

The ST4 genic sequence was heavily methylated at the CG and CHG sites in both the tomato and eggplant seed-plant controls and the self-grafted tomato (CG and CHG methylation levels being 80% and 61.9%, respectively), while less than 10% of the CHH sites were methylated (Figure 3A and B). In scions of the hetero-grafted plants (tomato grafted to eggplant, eT-1 and -2) methylation levels were increased at cytosine position surrounding the MSAP sites in all three sequence contexts (with an increase of 10–16% on CG methylation, 20% on CHG methylation and 10% on CHH methylation (Figure 3A and B). The result of bisulfite Sanger sequencing is consistent with the MSAP profile of this band (ST4) which was scored as CG-hypermethylation at the 5′-CCGG site(s) in the tomato scions (Table 2). In both progeny plants, the increased CG methylation was largely inherited while the increased CHG methylation was only partly inherited (Figure 3A and B). Further statistical test (see Methods for details) for methylation alterations in each of the self- and hetero-graft plants for each of the three sequence contexts, CG, CHG and CHH, as well as total Cs, relative to those in the seed-plant control, indicated that 13 of the 16 pairwise comparisons between hetero-grafted plants and control are highly significant (Table S5). Interestingly, this test also revealed that three of the four comparisons between self-grafted plants and control for this genic sequence are also significantly different, though to a much less extent than those of the hetero-grafts vs. control (Table S5). This suggests that self-grafting may also cause alterations in DNA methylation, which however are mild and can be revealed only by the single-base pair resolution analysis of locus-specific BS-Sanger sequencing.

The SE1 genic sequence is unmethylated in in both the eggplant and tomato seed-plant controls and the self-grafted eggplant, while scions of the hetero-grafted plants (eggplant grafted to tomato, tE-1 and −2) showed *de novo* methylation in all sequence contexts: CG (23% and 40%, respectively), CHG (15.5% and 34.5%, respectively), and CHH (5% and 7%, respectably) (Figure 3C and D). Furthermore, the *de novo* methylated sites was not only inherited to the progenies, but also showed further spreading into the surrounding regions (Figure 3C and D). The *de novo* methylation occurred in SE1 revealed by bisulfite sequencing is also consistent with its pattern alteration detected in the MSAP profile, in which it was scored as CHG-hypermethylation (Table 2). Further statistical analysis indicated that all 16 comparisons of methylation differences between the hetero-grafted plants and the seed-plant control are highly significant while none of the four comparisons between the self-grafts and control is significant for this genic sequence (Table S5).

The analyzed SE2 fragment represents a low-copy retrotransposon and contains only one CG site and one CHG site, which showed 33.3% and 100% methylation, respectively, in the eggplant seed-plant control and self-grafted eggplant (Figures 3c). The CHH methylation of this sequence is about 24% (Figures 3E and F), which is higher than in the two genic sequences described above (Figure 3A to D), and this feature is often characteristic of methylation in transposable elements. The CG methylation was increased to different extents in the two hetero-grafted (eggplant grafted to tomato) plant scions, tE-1, and −2, by 22.3% and 44.5%, respectively, while the CHG methylation remained unaltered in one scion plant (tE-1) but decreased by 44.4% in the other (tE-2) (Figure 3E and F). Interestingly, one progeny plant showed dramatic decrease in both CG and CHG methylation while the other progeny plant only showed decrease in CHG methylation (Figure 3E and F). The CHH methylation of this retrotransposon fragment showed a small extent of demethylation in the scion plants and/or their progenies as well (Figure 3c). The methylation alterations of SE2 revealed by bisulfite sequencing is also in broad agreement with its pattern alteration detected in the MSAP profile (Table 2). Further statistical analysis indicated that six of the 15 comparisons of methylation differences between the hetero-grafted plants and the seed-plant control are significant while two of the three comparisons between the self-grafts and control are significant (Table S5).

Taken together, all the three loci analyzed by bisulfite sequencing showed that the interspecific hetero-grafting has induced conspicuous alterations in DNA methylation involving cytosines of all sequence contexts, CG, CHG and CHH, while the self-grafting also caused mild changes in some of the methylation patterns. To a large extent, the altered methylation patterns in the scions of hetero-grafts are inherited to their selfed progenies. In addition, the methylation alteration patterns revealed by bisulfite sequencing are in agreement with those seen in the MSAP profiles, thus suggesting that the great majority, if not all, of the methylation-alterable loci detected in the MSAP profiles are authentic.

### Graft-induced perturbation in the steady-state transcript abundance of genes involved in establishing and maintaining of DNA methylation

Next, we investigated whether the graft-induced DNA methylation alterations would be accompanied by expression changes of genes associated with the DNA cytosine methylation establishment, maintenance or removal. We identified in the tomato genome the following putative genes by a functional search through the chromatin database (http://www.chromdb.org/): (1) three DNA methyltransferase-encoding genes, namely, *slMET1*, homologue of *Arabidopsis* methyltransferase 1 (*MET1*), which is responsible for the maintenance of CG methylation, *slCMT3*, homologue of *Arabidopsis* Chromomethylase 3 (*CMT3*), which is responsible for maintenance of CHG and CHH methylation, and *slDRM2*, homologue of *Arabidopsis* Domains-rearranged methyltransferase 2 (*DRM2*), which is a *de novo* DNA methylase responsible for establishment of cytosine methylation of all sequence contexts [32], [33]; (2) two active 5-methylcytosine DNA glycosylases encoding genes, *slROS1* and *slDME*, which are respectively homologues of *Arabidopsis ROS1* and *DME*, and responsible for active removal of excessively or aberrantly methylated cytosines in all sequence contexts [34], [35]; (3) four proteins involved in the biogenesis of siRNAs that participate in the RdDM pathway, that is, *slNRPDA*, homologue of *Arabidopsis NRPD1a* known as the largest subunit of PolIV (a plant specific RNA polymerase responsible for transcriptional dsRNA formation), *slRDR*, homologue of *Arabidopsis RDR2*, essential for the production of endogenous siRNAs, *slAGO*, homologue of *Arabidopsis AGO4*, important for dicing dsRNA into small RNAs, and *slDRB*, homologue of *Arabidopsis DRB2*, a double-strand RNA binding protein [26].

**Figure 4:**
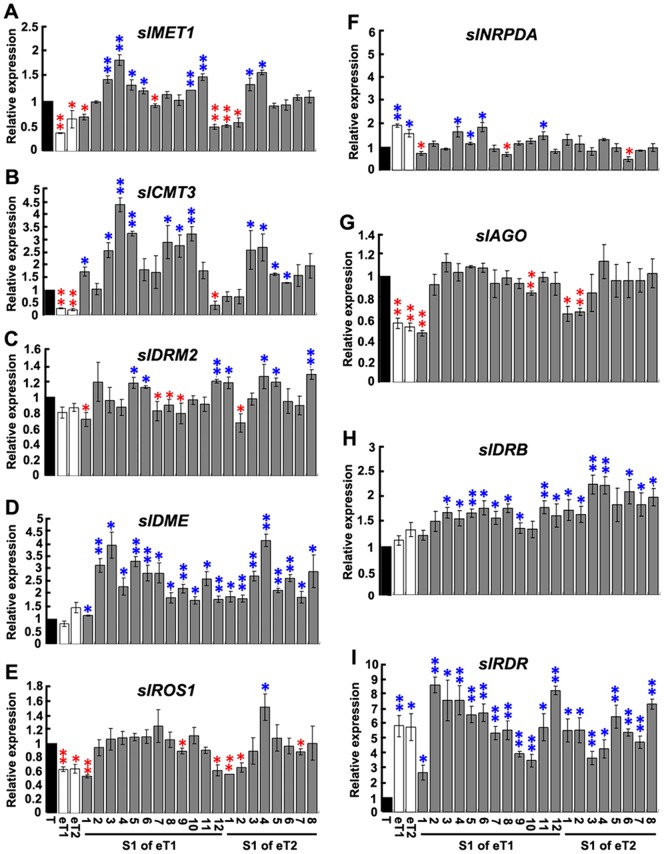
Real-time qRT-PCR analysis of genes encoding for enzymes involved in establishing and/or maintaining of DNA methylation. The steady-state transcript abundance of genes encoding three DNA methyltransferases, slMET1 (A), slCMT3 (B), and slDRM2 (C), two 5-methylcytosine DNA glycosylases, slDME (D) and slROS1 (E), and four proteins involved in the biogenesis of siRNAs participating in the RdDM pathway, slNRPDA (F), slAGO (G), slDRB (H), and slRDR (I) were analyzed by real-time qRT-PCR. All these genes were identified from the tomato genome (details are given in Table S1). Reverse transcription was performed on three batches of independently isolated RNAs from young leaves of the seed-plant control (T), two independent hetero-grafted (tomato to eggplant) scion plants (eT1-2), and 12 and eight selfed progeny plants (S1) of each mother scion plant. A conserved *Solanaceae* actin gene (Genbank accession: U60481) was used as an internal control for RNA input and DNA contamination (on RNAs without reverse transcriptase). Asterisks denote statistical difference in the transcript abundance relative to that of the corresponding seed-plant control, based on t-test at *P* = 0.05 (*) and *P* = 0.01 (**) levels, respectively. Colors of asterisks refer to significant up- (blue) and down-regulation (red), respectively.

We performed real-time, q-RT-PCR analysis for each of these genes in young-leaf tissue of two independent hetero-grafted (tomato to eggplant) tomato scion plants, their selfed progenies (from eight to 10 individuals), and seed-plant control. We observed that, relative to the seed-plant control, the transcript abundance of four genes, *slMET1*, *slCMT3*, *slROS1* and *slAGO*, were significantly reduced in both hetero-grafted scions, while in their selfed progenies, these four genes showed a common trend of up-regulation and returning back to their normal expression level similar to, or significantly higher than, the seed-plant control (Figure 4A, B, E and G). The transcript abundance of *slNRPD* and *slRDR* was increased in both hetero-grafted scions relative to the seed-plant control (Figure 4F and I). In the selfed progenies of these scions, one gene (*slNRPD*) showed a trend of down-regulation towards expression level of the control plant (Figure 4F) while the other gene (*slRDR*) showed a trend of persistent up-regulation (Figure 4I). For gene *slNRPD*, the selfed progenies displayed a range of expression levels when compared to the seed-plant control similar to those observed for *slNRPD*. Transcript abundance of the remaining three genes, *slDRM2*, *slDME* and *slDRB*, showed no significant difference in the hetero-grafted scions, but significant variations were observed in some of the progenies denoting protracted effect of hetero-grafting (Figure 4C, D and H).

## Discussion

Both historic and current agricultural practices testify that the effectiveness and utility of grafting in improving performance of horticultural plants is beyond doubt [10], [36]. The controversy concerns the existence and nature of “graft hybrids” [1], [25], [37]. Darwin is the first to put forth the terms “graft hybridization” and “graft hybrid” in one of his seminar books, *The Variation of Animals and Plants under Domestication*, in which he recorded various cases to substantiate his belief that grafted organismal products may contain properties of, and hence are genetically distinct from, the grafting partners [11]. Nonetheless, the graft hybrid concept has gained a nearly consensus denial in the scientific community due to the lack of a sound mechanistic basis to explain this phenomenon [37].

The recently documented mobility of various genetic components including DNAs, RNAs and proteins between the scion and stock, at least within the immediate grafting junctions, have unequivocally proven that not only phenotypic traits but also the core molecular building blocks could be altered in the grafted products [10]. Thus, the term graft hybrid is correct in the sense that the grafted plants can be genetically distinct from both “parental” grafting partners. Nonetheless, the exchange of plastid genomic fragment or the whole plastid genome so far documented occurred at very low frequencies, which entailed antibiotic selections to identify [22]–[24]. On the other hand, improved agronomic performance of the grafted plant is expected to emerge homogeneously in the whole grafted scions, a feature essential for its practical utility. Apart from the physiological and biochemical aspects of grafting (e.g., exchanges in nutrients, hormones etc.), the uniformly changed phenotypes might have a heritable basis, which would imply the existence of more generally occurring underpinnings. This said, an automatic reflection would be an epigenetics-based mechanism, as which can arise rapidly and be independent of the low-incidence, if any, genetic exchanges and/or alterations [10].

The finding in *Arabidopsis* that a substantial proportion of the endogenous small RNAs of all size classes (21–24 nt) can move across the graft union via plasmodesmata and phloem, and in particular the moved 24-nt siRNAs was capable of directing DNA methylation in the genome of the recipient cells [29] is tantalizing, as it has provided a solid molecular basis for grafting-induced phonotypical changes. We have shown in this study that although the relative *collective* level of DNA methylation remained largely unaffected, extensive alterations in at least four DNA methylation patterns, CG hypo, CG hyper, CHG hypo and CHG hyper of the randomly sampled 5′-CCGG sites, which can be assessed by the MSAP marker, occurred in all independent samples of multiple interspecific graftings tested involving three *Solanaceae* species. This unprecedented extent of unanimous methylation changes strongly suggested generality of the graft-induced epigenetic modification phenomenon. It should be emphasized that both methylation alterations and their heritability were validated by bisulfite sequencing at all three investigated loci. Notably, in some cases, significant alteration in DNA methylation pattern was also detected by the bisulfite sequencing analysis in the self-grafted plants, although which was not detected by the less-sensitive MSAP analysis. Together, it is clear that hetero-grafting represents a potent condition to generate DNA methylation alterations, and the induced epigenetic modifications could have affected the primordial cells that are destined to form gametal cells.

The dramatic nature with regard to the extensiveness of the methylation pattern alterations would intuitionally calls for concerns on the genetic purity or homogeneity of the plants used for the grafting manipulations. As described in the Material & Method section, all plants of the three *Solanaceae* species, tomato, potato and pepper are pure-line cultivars that have been maintained for many generations by strict selfing at the Jilin Academy of Vegetables and Flowers, Changchun, China. Therefore, both genetic and epigenetic heterozygosity in these lines should be minimal. To further verify this, we performed a MSAP assay on a subset of 15 randomly selected individuals from each of the three cultivar used for the grafting experiments with the same primer combinations as used for analyzing the grafted plants (Table S1). We found that if one plant individual was arbitrarily taken as a “control” to tabulate the rest individuals, alteration frequencies for all the four methylation patterns (CG hypo, CG hyper, CHG hypo and CHG hyper) were nonexistent to very low, rendering the alteration frequencies for the total methylation patterns (adding up the four patterns) being lower than 1.5% (data not shown). This is in sharp contrast to the extensively altered methylation patterns for each of the the four types in the hetero-grafted plants (Figure 2), which would exceed 15% for the total methylation patterns (adding up the four patterns). Thus, the extensive methylation pattern alterations detected in the grafted scions or rootstocks should have been primarily induced by hetero-grafting.

Given the important biological roles played by DNA methylation in plants, it is reasonable to suspect that the extensively altered methylation patterns may produce functional consequences. This possibility is bolstered by the results that of the methylation-altered genomic loci, genic sequences were substantially enriched, and which involved genes of diverse biological functions. Together with their general occurrence, it is tempting to propose that DNA methylation alteration likely constitutes an important genetic component underlying the Darwinian concepts of graft hybridization and graft hybrid. It is remarkable that grafting as an ancient practice with enormous practical value is likely being intrinsically related to epigenetic phenomena.

Our observation that the DNA methylation pattern alterations in the grafts are concomitant with perturbed expression of a set of chromatin-regulation genes implies a possible source for their genesis. It is well-established in both animals and plants that the level and pattern of cytosine methylation are established and perpetuated by a diverse set of chromatin- regulation proteins that act both synergistically and antagonistically [38]–[41]. We have observed that, relative to the control, the nine studied chromatin-regulation genes did show perturbed expression in the immediately grafted scions and/or their selfed progenies. These studied genes included those known to function directly to establish, maintain and remove cytosine methylation, and those involved in the biogenetics of the 24 nt siRNAs that are involved in the RdDM pathway [26]–[28], [42]. Therefore, it is likely that the methylation pattern alterations and their inheritance induced by grafting are at least in part due to perturbed expression of the cellular machinery required for DNA methylation, which warrants further investigations that may entail the employment of various mutants for each and combinations of these chromatin-regulation genes.

To conclude, our results have demonstrated that inter-species hetero-grafting, at least in the *Solanaceae* plants, could produce heritable alteration in DNA cytosine methylation patterns. We suggest that epigenetic modifications, rather than genetic changes, might be the molecular underpinnings for the still contentious concept of graft hybridization and graft hybrid.

## Materials and Methods

### Plant materials

Pure-line cultivars of three species of *Solanaceae*, tomato (*Solanum lycopersicum* L.) cv. BF101, eggplant (*Solanum melongena* L.) cv. B42, and pepper (*Capsicum annuum* L.) cv. P035, designated as T, E, and P, respectively, were used in this study. Tomato and eggplant were reciprocally grafted onto each other, and the two kinds of scions were used for study. Pepper was used for study only as the rootstock of tomato. All cultivars were maintained by strict self-pollination at Jilin Academy of Vegetables and Flowers, Changchun 130033, China.

### Grafting

Grafting of soil-grown plants was performed in greenhouse. Seedlings were grown from germinating seeds, and they reached the stage with 6–8 real leaves, the tomato and eggplant plants as scions were prepared by making a V-shape cut on the exposed end of the trimmed shoot apex (retaining 2–3 real leaves). Plants as rootstocks for all the grafts were prepared by decapitating the shoot apex from about 10 cm above the soil, and 1.0–1.5 cm deep slits were made. Scions were inserted into the slits of the rootstock, and the pots were immediately enclosed in transparent polyethylene bags. Grafts were maintained and gradually hardened in the greenhouse for about two weeks when the unions were strengthened and plants reached the 8–10-leaf stage. A total avoidance of sunlight was conducted on the graft unions for the first week subsequent to the grafting manipulations, and then the shading of light was gradually reduced. After 10 days of grafting, a normal light condition (sunlight) was introduced during daytime.

### MSAP analysis

Genomic DNA was isolated from expanded leaves of the control seed-plants, three independent scion/rootstock plants for each species, and the S1 progeny individuals (Table S6), by the high-salt CTAB protocol [43]. Methylation-sensitive amplified polymorphism (MSAP) is a modified version of AFLP (amplified fragment length polymorphism) marker by incorporating methylation-sensitive restriction enzymes, and was proven to be an efficient technique to detect DNA methylation alterations at randomly sampled loci from a genome-wide perspective [31]. The detailed strategy for the primers and adaptors design, and experimental procedure was described previously[44]. In the study, we used a pair of isoschizomers, *Hpa*II and *Msp*I, for MSAP. This pair of isoschizomers recognize the same tetra-nucleotide sequence, 5′-CCGG, but have different sensitivities to methylation of the cytosines: *Hpa*II will not cut if either of the cytosines in the double strand is methylated, whereas *Msp*I will not cut if the external cytosine is fully- or hemi- (single-strand) methylated. Thus, for a given DNA sample in the MSAP profiles, full methylation of the internal cytosine (designated as CG full methylation) or hemi-methylation of the external cytosine (designated as CHG hemi-methylation) at the assayed 5′-CCGG sites is revealed as the presence of a band in only one of the enzyme digests and absence from the other; on the other hand, simultaneous hyper- or hypo-methylation of both cytosines will be revealed as the concomitant absence or presence of a band with both enzymes relative to the sample of the same genotype with unmethylated cytosines at the 5′-CCGG site in question (Table S3). To simplify the scoring, we divided the MSAP patterns into four major types: (1) CG hypermethylation (summary of A3, A4 and B4 in Table S3); (2) CHG hypermethylation (summary of A2, A4 and C4 in Table S3); (3) CG hypomethylation (summary of C2, D2 and D3 Table S3), and; (4) CHG hypomethylation (summary of B2, D2 and D4 Table S3). In this study, one pair of pre-selective primers and 25 pairs of selective primers were used (Table S1A). The amplification products of MSAP were resolved by 5% denaturing polyacrylamide gel electrophoresis and visualized by silver staining. Two technical replicates were conducted (starting from DNA digestion, i.e., the first step in MSAP). Only those clear and reproducible bands that appeared in two independent PCR amplifications (starting from the digestion-ligation step, i.e., the first step of MSAP) were scored.

### Statistical analysis for MSAP

Statistical analysis for the methylation level differences between the control plants and the grafted plants was performed as previously described [45]. Briefly, the scored MSAP bands were transformed into a binary character matrix, “1” for presence and “0” for absence of a band, at a particular position. The U test was performed to investigate differences between the seed-plants value and the grafted grafted scions/rootstocks. The following formula was used:
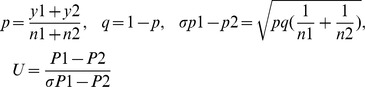
 where n1 is the total sites (of CCGG) we detected for a given sample (herein refers to the seed-plant values); n2 the total sites we detected for another sample (herein refers to a grafted scions/rootstock); y1 is the total methylation sites, hemi-methylation sites (CHG) or full methylation sites (CG) for a given sample (herein refers to the seed-plant values); y2 is the total methylation sites, hemi-methylation sites or full methylation sites for another sample (herein refers to a grafted scions/rootstock); p1 the percentage of total methylation sites, hemi-methylation sites or full methylation sites for a given sample (herein refers to the seed-plant values); p2 is the percentage of total methylation sites, hemimethylation sites or fully methylation sites for another sample (herein refers to a grafted scions/rootstock).

### Recovery and sequencing of MSAP bands

A subset of bands showing various patterns of DNA methylation alterations in grafted (both scion and rootstock) plants relative to the control seed-plants were eluted from the silver-stained MSAP gels by 70°C water-bath incubating for 90 min in 20 µl 1xTE buffer, and re-amplified using the same tracing selective primer combinations. After the sizes of the PCR products were verified by electrophoresis, they were gel purified and cloned into the pMD18-T vector (Takara Biotechnology Inc.) and were sent to Sangon Biotech (Shanghai) Co. Ltd. for sequencing. The Advanced BLASTN and BLASTX programs of the NCBI (http://www.ncbi.nlm.nih.gov/) were used for sequence and functional homology analysis of the cloned DNA bands that gave quality reads.

### Bisulfite Sanger sequencing

Genomic DNA was modified using an EZ DNA Methylation-Gold kit (Zymo Research) according to the manufacturer's recommendations. Briefly, 900 µl dd H_2_O, 50 µl M-dissolving buffer and 300 µl M-dilution buffer (Zymo Research) were added per tube of C-T conversion reagent (Zymo Research) prior to use. Then 130 µl of bisulfite containing C-T conversion reagent was added to 1 µg of DNA in a volume of 20 µl (150 µl in total) and thoroughly mixed, and the samples were incubated at 98°C for 10 min, and 64°C for 2.5 h. Modified DNA was purified using a Zymo-Spin IC column (Zymo Research) and stored at −20°C until use. For each PCR reaction, 3.0 µl of bisulfite treated DNA was used in a 10 µl reaction system and the PCR products were cloned into the pMD18-T vector and sequenced. The condition for the polymerase chain reaction was: 94°C for 2 min; 12 cycles of 94°C for 30 sec, 65°C for 30 sec (1°C decrease for each cycle), and 72°C for 80 sec; 25 cycles of 94°C for 30 sec, 55°C for 30 sec, and 72°C for 80 sec; 72°C for 10 min. Ten to 20 clones were sequenced for each sample. The primers for two unique-copy genic sequences (ST4 and SE1, respectively from tomato and eggplant) and a low-copy retrotransposon (SE2 from eggplant) for bisulfite sequencing were designed using the Meth-Primer program (http://www.urogene.org/methprimer/) and are given in Table S1B. The methylation levels expressed as percentage (%) per site for each of the three types of cytosines, CG, CHG and CHH, were calculated by dividing the number of non-converted (methylated) cytosines by the total number of cytosines of each type within the sequenced regions. The methylation level of each cytosine locus for each single colony of these plant samples was revealed using the Kismeth program (http://katahdin.mssm.edu/kismeth/revpage.pl). Chi-squared test was conducted for statistic difference analysis between the seed-plant control and each of of the self- and hetero-graft plants in each of the three sequence contexts, CG, CHG and CHH, as well as total.

### Quantitative RT-PCR analysis

The real-time q-RT-PCR analysis was only conducted on tomato because of its available genome information. Total RNA was isolated from young leaves of seed-plant controls, two individuals of the hetero-grafted (tomato to eggplant) scion plants, and their S1 and S2 progeny individuals by the Trizol Reagent (Invitrogen) according to manufacturer's protocol. For q-RT-PCR, the RNA was further treated with DNase I (Invitrogen), and reverse transcribed by the SuperScript RNase H^−^Reverse Transcriptase (Invitrogen). 120 µl TE buffer was added to the cDNA products of each reverse transcription of 5 µg RNA, and 0.5 µl cDNA sample was added to 2×SYBR Green Real-time PCR Master Mix (Code: QPK-201, TOYOBO CO., LTD.) for one reaction, which was conducted on Roche LightCycler 480 Instrument (Roche Applied Science, Germany). Three independent reactions were repeated and 2^−ΔΔCT^ method was used for the result analysis. The primers for the *Solanaceae* universal actin gene (Genbank accession: U60481) was used as an internal control. Three putative DNA methyltransferase encoding genes, two putative 5-methylcytosine DNA glycosylase encoding genes and four putative genes encoding for enzymes involved in the RdDM pathway were designed by using the Primer Premier 5 software and given in Table S1C. For statistical analysis of q-RT-PCR, we performed the t-test to confirm if any significant difference occurred between seed-plants and grafted-plants. Statistical significance was determined using SPSS 11.5 for Windows (http://www.spss.com/st atistics/) and analyzed by Student's *t*-test.

## Supporting Information

Figure S1 **Tabulated data of relative DNA methylation levels and alteration patterns in rootstock based on the MSAP analysis.** (**A**) Relative levels of total methylation, CG methylation and CHG methylation at randomly 5′-CCGG sites in three independent hereto-grafted pepper rootstocks (scioned by tomato, marked as Pt1-3), and the pepper seed-plant control (P). (**B**) Alterations in the four major methylation patterns, CG hyper, CHG hyper, CG hypo and CHG hypo, in the three independent rootstock peppers (tP1-3), was calculated relative to the pepper seed-plant control.
(TIF)

Table S1 **Sequences of adaptors and primers for MSAP analysis, and primers for bisulfite sequencing and real-time qRT-PCR.**
(DOC)

Table S2 **Levels of DNA methylation at the randomly sampled 5′-CCGG sites by MSAP in hetero-grafted plants (scions and rootstocks) and their corresponding seed-plant controls.**
(DOC)

Table S3 **Patterns of DNA methylation at the randomly sampled 5′-CCGG sites and their frequencies in the hetero-grafted plants.**
(DOC)

Table S4 **Sequences and analysis of variant MSAP bands isolated from hetero-grafted scions and rootstocks.**
(DOC)

Table S5 **Chi-squared test for statistical significance in frequencies of methylated cytosines** ***vs.*** **total cytosines (based on BS-seq) in each of the three sequence contexts, CG, CHG and CHH, as well as total C for each of the three analyzed sequences, between the seed-plant control and each of the self- and hetero-grafted plants by using R package.**
(DOC)

Table S6 **Details of the grafted plants used in this study.**
(DOC)
