# Supplementary figures and images for: Inter-Species Grafting Caused Extensive and Heritable Alterations of DNA Methylation in Solanaceae Plants
Source: PLoS One. 2013 Apr 16;8(4):e61995. doi: 10.1371/journal.pone.0061995 (PMC3628911; doi:10.1371/journal.pone.0061995)

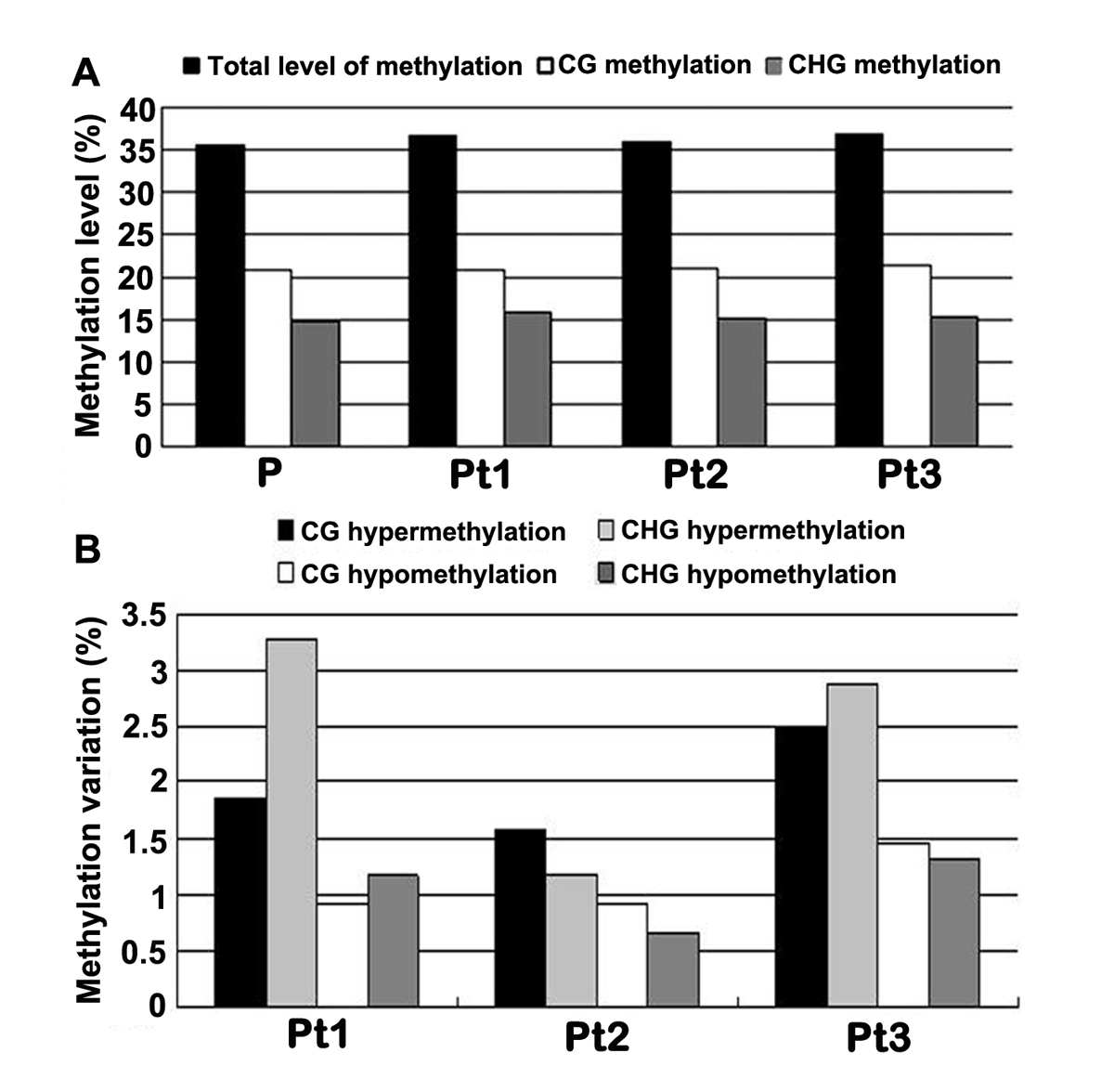

Supplement: Figure S1 — Tabulated data of relative DNA methylation levels and alteration patterns in rootstock based on the MSAP analysis. (A) Relative levels of total methylation, CG methylation and CHG methylation at randomly 5′-CCGG sites in three independent hereto-grafted pepper rootstocks (scioned by tomato, marked as Pt1-3), and the pepper seed-plant control (P). (B) Alterations in the four major methylation patterns, CG hyper, CHG hyper, CG hypo and CHG hypo, in the three independent rootstock peppers (tP1-3), was calculated relative to the pepper seed-plant control. (TIF) [file pone.0061995.s001.tif]
