# Supplementary material for: Inter-Species Grafting Caused Extensive and Heritable Alterations of DNA Methylation in Solanaceae Plants
Source: PLoS One. 2013 Apr 16;8(4):e61995. doi: 10.1371/journal.pone.0061995 (PMC3628911; doi:10.1371/journal.pone.0061995)
Supplement: Table S1 — Sequences of adaptors and primers for MSAP analysis, and primers for bisulfite sequencing and real-time qRT-PCR. (DOC) [file pone.0061995.s002.doc]

**Table S1.** Sequences of adaptors and primers for MSAP analysis, and primers for bisulfite sequencing and real-time qRT-PCR.

| **Adaptors and primers for MSAP** | |  |
| --- | --- | --- |
| **Adapters** | |  |
| *Eco*RI-adapter I | 5'-CTCGTAGACTGCGTACC-3' |  |
| *Eco*RI-adapter II | 5'-AATTGGTACGCAGTC-3' |  |
| H/M-adapter I | 5'-GATCATGAGTCCTGCT-3' |  |
| H/M-adapter II | 5'-CGAGCAGGACTCATGA-3' |  |
| **^†^Pre-selective primers** | |  |
| E-A | 5'-GACTGCGTACCAATTCA-3' |  |
| H/M-0 | 5'-ATCATGAGTCCTGCTCGG-3' |  |
| **Selective primers** | |  |
| H/M primers | |  |
| 1. H/M-TCT | 5'-ATCATGAGTCCTGCTCGGTCT-3' |  |
| 2. H/M-TCG | 5'-ATCATGAGTCCTGCTCGGTCG-3' |  |
| 3. H/M-TCC | 5'-ATCATGAGTCCTGCTCGGTCC-3' |  |
| 4. H/M-TTC | 5'-ATCATGAGTCCTGCTCGGTTC-3' |  |
| 5. H/M-TTG | 5'-ATCATGAGTCCTGCTCGGTTG-3' |  |
| 6. H/M-TTA | 5'-ATCATGAGTCCTGCTCGGTTA-3' |  |
| *Eco*RI primers | |  |
| b. E-AAG | 5'-GACTGCGTACCAATTCAAG-3' (combined with 4, 5 H/M primers) |  |
| c. E-ACA | 5'-GACTGCGTACCAATTCACA-3' (combined with 3, 4, 5, 8 and 10 H/M primers) |  |
| d. E-ACT | 5'-GACTGCGTACCAATTCACT-3' (combined with 4, 6, 7 and 9 H/M primers) |  |
| e. E-ACC | 5'-GACTGCGTACCAATTCACC-3' (combined with 4, 6 H/M primers) |  |
| g. E-AGC | 5'-GACTGCGTACCAATTCAGC-3' (combined with 1, 4 H/M primers) |  |
| h. E-AGG | 5'-GACTGCGTACCAATTCAGG-3' (combined with 1, 3, 4, 5, 6 and 10 H/M primers) |  |
| i. E-AGA | 5'-GACTGCGTACCAATTCAGA-3' (combined with 4, 5, 6 and 8 H/M primers) |  |
| **Primers for bisulfite sequencing** | |  |
| **MSAP bands** | **Primer sequence* Product size（bp）** |  |
| ST4 | Forward: 5’- agttayyayagggataaytggyttgtggyag 336 |  |
|  | Reverse: 5’- ctarcccrrattctracttararrcrttca |  |
| SE1 | Forward: 5’- yggttygggtatttytgaagtayaaagggg 212 |  |
|  | Reverse: 5’- rrrrtarrrtactacraaarraarttratc |  |
| SE2 | Forward: 5’- yggttgtyataattgyyaayaagtgaaagyyaa 186 |  |
|  | Reverse: 5’- tacaacttracataatactcrrtaccataa |  |
| **Primers for real-time qRT-PCR** | |  |
| **Gene name** | **Primer sequence Product Sequence sources and its** |  |
|  | **size (bp) homology to *Arabidopsis*** |  |
| *Actin* | Forward: 5’- tggatttgctggtgatgatg 133 Accession: U60481 (Genbank) |  |
|  | Reverse: 5’- acccctcttggattgagctt |  |
| *slMET1* | Forward: 5’- accagcatagaaacacg 144 Accession: AJ002140 (Genebank) |  |
|  | Reverse: 5’- tcttatcaaacgggagt Homologous to MET1 of *Arabidopsis* |  |
| *slCMT3* | Forward: 5’- agaacaatgagccaagag 205 Accession: TA38631 (Tigr) |  |
|  | Reverse: 5’- tcaactgaagaggacgat Homologous to CMT3 of *Arabidopsis* |  |
| *slDRM2* | Forward: 5’- tgagcctgagtttgtcg 115 Accession: EU344815 (Genbank) |  |
|  | Reverse: 5’- ggtgcgtggaggaagt Homologous to DRM2 of *Arabidopsis* |  |
| *slROS1* | Forward: 5’- gataccatgccgaacagc 134 Accession: TA47202 (Tigr) |  |
|  | Reverse: 5’- ggagattccacagccaat Homologous to ROS1 of *Arabidopsis* |  |
| *slDME* | Forward: 5’- gaacgagggatgaacaac 146 Accession: BG123849 (Tigr) |  |
|  | Reverse: 5’- agtccacgaatactcaaaa Homologous to DME of *Arabidopsis* |  |
| *slRDR* | Forward: 5’- gatgggttggtgtatgtaag 104 Accession: RDR1101 (ChromDB) |  |
|  | Reverse: 5’- tcagaataattgcggaga Homologous to RDR2 of *Arabidopsis* |  |
| *slNRPDA* | Forward: 5’- taagggcaacctgctaaa 215 Accession: NRPDA1101 (ChromDB) |  |
|  | Reverse: 5’- aacccgcaagaaatgaac Homologous to NRPD1a of *Arabidopsis* |  |
| *slAGO* | Forward: 5’- tacaactcgtcctactcact 153 Accession: AGO1101 (ChromDB) |  |
|  | Reverse: 5’- gcaagatgggcataaca Homologous to AGO4 of *Arabidopsis* |  |
| *slDRB* | Forward: 5’- ttccgacacctaatcca 247 Accession: DRB1101 (ChromDB) |  |
|  | Reverse: 5’- cgaaccctgtgactatcc Homologous to DRB2 of *Arabidopsis* |  |

**^†^**H/M: abbreviation of *Hpa*II/*Msp*I; E: abbreviation of *Eco*RI.

* r=a/g；y=c/t.
