# Supplementary material for: Inter-Species Grafting Caused Extensive and Heritable Alterations of DNA Methylation in Solanaceae Plants
Source: PLoS One. 2013 Apr 16;8(4):e61995. doi: 10.1371/journal.pone.0061995 (PMC3628911; doi:10.1371/journal.pone.0061995)
Supplement: Table S2 — Levels of DNA methylation at the randomly sampled 5′-CCGG sites by MSAP in hetero-grafted plants (scions and rootstocks) and their corresponding seed-plant controls. (DOC) [file pone.0061995.s003.doc]

**Table S2.** Levels of DNA methylation at sampled 5’-CCGG sites by MSAP in hetero-grafted plants (rootstocks and scions) and seed-plant controls.

| Plant sample | | Total sites | Unmethylated sites | Methylated sites | | |
| --- | --- | --- | --- | --- | --- | --- |
|  | |  |  | CG full methylation | CHG hemi-  methylation | Total |
| Seed plant | P | 761 | 490 (64.39%) | 158.5 (20.83%) | 112.5 (14.78%) | 271 (35.61%) |
|  | T | 843 | 562 (66.67%) | 177 (21.00%) | 104 (12.33%) | 281 (33.33%) |
|  | E | 764 | 507 (66.36%) | 170 (22.25%) | 87 (11.39%) | 257 (33.64%) |
| Scion | eT1 | 843 | 578 (68.56%) | 165.5 (19.64%) | 99.5 (11.80%) | 265 (31.44%) |
|  | eT2 | 843 | 599 (71.06%) | 151 (17.91%)* | 93 (11.03%) | 244 (28.94%)* |
|  | eT3 | 843 | 571 (67.73%) | 174.5 (20.70%) | 97.5 (11.57%) | 272 (32.27%) |
|  | tE1 | 764 | 519 (67.93%) | 160 (20.94%) | 85 (11.13%) | 245 (32.07%) |
|  | tE2 | 764 | 516 (67.54%) | 160.5 (21.01%) | 87.5 (11.45%) | 248 (32.46%) |
|  | tE3 | 764 | 513 (67.15%) | 163 (21.33%) | 88 (11.52%) | 251 (32.85%) |
| Rootstock | Pt1 | 761 | 482 (63.34%) | 158 (20.76%) | 121 (15.90%) | 279 (36.66%) |
|  | Pt2 | 761 | 487 (63.99%) | 159.5 (20.96%) | 114.5 (15.05%) | 274 (36.01%) |
|  | Pt3 | 761 | 481 (63.21%) | 163 (21.42%) | 117 (15.37%) | 280 (36.79%) |

*Difference is significant at the 0.05 statistical levels (two-tailed).

P, T and E are control seed-plants of potato, tomato and pepper, respectively; eT1-3 are independent hetero-grafted (tomato to eggplant) tomato scions; tE1-3 are independent hetero-grafted (eggplant to tomato) eggplant scions; Pt1-3 are independent hetero-grafted pepper rootstocks(scioned by tomato);
