# Supplementary material for: Inter-Species Grafting Caused Extensive and Heritable Alterations of DNA Methylation in Solanaceae Plants
Source: PLoS One. 2013 Apr 16;8(4):e61995. doi: 10.1371/journal.pone.0061995 (PMC3628911; doi:10.1371/journal.pone.0061995)
Supplement: Table S3 — Patterns of DNA methylation at the randomly sampled 5′-CCGG sites and their frequencies in the hetero-grafted plants. (DOC) [file pone.0061995.s004.doc]

**Table S3.** Patterns of cytosine methylation at 5'- CCGG sites and their frequencies in hetero-grafted plants.

|  | Seed-plant control | | | | Hetero-grafted plants | | | | | | | | |
| --- | --- | --- | --- | --- | --- | --- | --- | --- | --- | --- | --- | --- | --- |
|  | H^*^ | M | H | M | Number and frequency (%) | | | | | | | | |
|  |  |  |  |  | Rootstocks | | | Scions | | | | | |
|  |  |  |  |  | Pt1 | Pt2 | Pt3 | eT1 | eT2 | eT3 | tE1 | tE2 | tE3 |
| A1 | + | + | + | + | 473 (62.16) | 481 (63.21) | 469 (61.63) | 530 (62.87) | 527 (62.51) | 526 (62.40) | 487 (63.74) | 482 (63.09) | 481 (62.96) |
| A2 | + | + | + | - | 8 (1.05) | 3 (0.39) | 6 (0.79) | 6 (0.71) | 6 (0.71) | 5 (0.59) | 3 (0.39) | 2 (0.26) | 2 (0.26) |
| A3 | + | + | - | + | 3 (0.39) | 6 (0.79) | 12 (1.58) | 10 (1.19) | 12 (1.42) | 13 (1.54) | 12 (1.57) | 20 (2.62) | 20 (2.62) |
| A4 | + | + | - | - | 6 (0.79) | 0 (0) | 3 (0.39) | 10 (1.19) | 11 (1.30) | 12 (1.42) | 5 (0.65) | 3 (0.39) | 4 (0.52) |
| Total |  |  |  |  | 490 (64.39) | 490 (64.39) | 490 (64.39) | 556 (65.95) | 556 (65.95 | 556 (65.95) | 507 (66.36) | 507 (66.36) | 507 (66.36) |
| B1 | + | - | + | - | 95 (12.48) | 98 (12.88) | 96 (12.61) | 65 (7.71) | 64 (7.59) | 63 (7.47) | 51 (6.68) | 50 (6.54) | 51 (6.68) |
| B2 | + | - | + | + | 5 (0.66) | 2 (0.26) | 4 (0.53) | 6 (0.71) | 7 (0.83) | 6 (0.71) | 6 (0.79) | 6 (0.79) | 5 (0.65) |
| B3 | + | - | - | + | 1 (0.13) | 0 (0) | 2 (0.26) | 0 (0) | 0 (0) | 1 (0.12) | 1 (0.13) | 3 (0.39) | 1 (0.13) |
| B4 | + | - | - | - | 5 (0.66) | 6 (0.79) | 4 (0.53) | 2 (0.24) | 2 (0.24) | 3 (0.36) | 2 (0.26) | 1 (0.13) | 3 (0.39) |
| Total |  |  |  |  | 106 (13.93) | 106 (13.93) | 106 (13.93) | 73 (8.66) | 73 (8.66) | 73 (8.66) | 60 (7.85) | 60 (7.85) | 60 (7.85) |
| C1 | - | + | - | + | 137 (18.00) | 142 (18.66) | 132 (17.35) | 131 (15.54) | 119 (14.12) | 132 (15.66) | 128 (16.75) | 125 (16.36) | 128 (16.75) |
| C2 | - | + | + | + | 3 (0.39) | 3 (0.39) | 7 (0.92) | 15 (1.78) | 25 (2.97) | 13 (1.54) | 6 (0.79) | 11 (1.44) | 6 (0.79) |
| C3 | - | + | + | - | 1 (0.13) | 1 (0.13) | 0 (0) | 0 (0) | 1 (0.12) | 0 (0) | 1 (0.13) | 1 (0.13) | 2 (0.26) |
| C4 | - | + | - | - | 11 (1.45) | 6 (0.79) | 13 (1.71) | 16 (1.90) | 17 (2.02) | 17 (2.02) | 8 (1.05) | 6 (0.79) | 7 (0.92) |
| Total |  |  |  |  | 152 (19.97) | 152 (19.97) | 152 (19.97) | 162 (19.22) | 162 (19.22) | 162 (19.22) | 143 (18.72) | 143 (18.72) | 143 (18.72) |
| D1 | - | - | - | - | 6 (0.79) | 7 (0.92) | 4 (0.53) | 15 (1.78) | 2 (0.24) | 13 (1.54) | 13 (1.70) | 3 (0.39) | 4 (0.52) |
| D2 | - | - | + | + | 1 (0.13) | 1 (0.13) | 1 (0.13) | 27 (3.20) | 40 (4.74) | 26 (3.08) | 20 (2.62) | 17 (2.23) | 21 (2.75) |
| D3 | - | - | + | - | 3 (0.39) | 3 (0.39) | 3 (0.39) | 7 (0.83) | 6 (0.71) | 7 (0.83) | 16 (2.09) | 28 (3.66) | 24 (3.14) |
| D4 | - | - | - | + | 3 (0.39) | 2 (0.26) | 5 (0.66) | 3 (0.36) | 4 (0.47) | 6 (0.71) | 5 (0.65) | 6 (0.79) | 5 (0.65) |
| Total |  |  |  |  | 13 (1.71) | 13 (1.71) | 13 (1.71) | 52 (6.17) | 52 (6.17) | 52 (6.17) | 54 (7.07) | 54 (7.07) | 54 (7.07) |
| Sum |  |  |  |  | 761 (100%) | 761 (100%) | 761 (100%) | 843 (100%) | 843 (100%) | 843 (100%) | 764 (100%) | 764 (100%) | 764 (100%) |

Pt1-3 are independent hetero-grafted pepper rootstocks (scioned by tomato); eT1-3 are independent hetero-grafted (tomato to eggplant) tomato scions; tE1-3 are independent hetero-grafted (eggplant to tomato) eggplant scions;.

*the previous H and M representing band patterns of *Hpa*II- and *Msp*I-digest in control and the latter representing these in the self- and hetero-graft plants.
