# Supplementary material for: Inter-Species Grafting Caused Extensive and Heritable Alterations of DNA Methylation in Solanaceae Plants
Source: PLoS One. 2013 Apr 16;8(4):e61995. doi: 10.1371/journal.pone.0061995 (PMC3628911; doi:10.1371/journal.pone.0061995)
Supplement: Table S4 — Sequences and analysis of variant MSAP bands isolated from hetero-grafted scions and rootstocks. (DOC) [file pone.0061995.s005.doc]

**Table S4** . Grafting-induced variant MSAP fragments isolated from scions or rootstocks

| Clone ID | species originated | Blast-based homology | | Length of the sequences |
| --- | --- | --- | --- | --- |
|  |  | Hit of blastX | Hit of blastN |  |
| RP2 | pepper | Phosphoserine aminotransferase [*Arabidopsis thaliana*] [gb|AAM64881.1|](http://www.ncbi.nlm.nih.gov/entrez/query.fcgi?cmd=Retrieve&db=Protein&list_uids=21592931&dopt=GenPept&RID=W7Z2Z90F013&log$=protalign&blast_rank=2) | *Solanum lycopersicum* Tomato chromosome 2, C02SLe0031D11, complete sequence [gb|AC215451.1|](http://www.ncbi.nlm.nih.gov/entrez/query.fcgi?cmd=Retrieve&db=Nucleotide&list_uids=161789438&dopt=GenBank&RID=W9FBUUSE012&log$=nuclalign&blast_rank=1) | 168 |
| RP3 | pepper | Disease resistance-responsive family protein / fibroin-related [*Arabidopsis thaliana*] [ref|NP_180435.1|](http://www.ncbi.nlm.nih.gov/entrez/query.fcgi?cmd=Retrieve&db=Protein&list_uids=15226923&dopt=GenPept&RID=W7Z2Z90F013&log$=protalign&blast_rank=5) | *Arabidopsis thaliana* disease resistance-responsive family protein / fibroin-related (AT2G28670) mRNA, complete cds [ref|NM_128428.4|](http://www.ncbi.nlm.nih.gov/entrez/query.fcgi?cmd=Retrieve&db=Nucleotide&list_uids=145360428&dopt=GenBank&RID=W9FBUUSE012&log$=nuclalign&blast_rank=3) | 410 |
| RP4 | pepper | PHO1-like protein [*Arabidopsis thaliana*] [gb|AAR99488.1|](http://www.ncbi.nlm.nih.gov/entrez/query.fcgi?cmd=Retrieve&db=Protein&list_uids=41079275&dopt=GenPept&RID=W7Z2Z90F013&log$=protalign&blast_rank=2) | *Arabidopsis thaliana* EXS family protein / ERD1/XPR1/SYG1 family protein(AT2G03250) mRNA, complete cds [ref|NM_126376.2|](http://www.ncbi.nlm.nih.gov/entrez/query.fcgi?cmd=Retrieve&db=Nucleotide&list_uids=42568899&dopt=GenBank&RID=W9FBUUSE012&log$=nuclalign&blast_rank=3) | 110 |
| RP5 | pepper | Kunitz-type proteinase inhibitor precursor [*Solanum tuberosum*] [gb|AAN04125.1|](http://www.ncbi.nlm.nih.gov/entrez/query.fcgi?cmd=Retrieve&db=Protein&list_uids=22655597&dopt=GenPept&RID=W7Z2Z90F013&log$=protalign&blast_rank=1) |  | 245 |
| RP10 | pepper |  | *Nicotiana tabacum* mitochondrial DNA, complete genome [dbj|BA000042.1|](http://www.ncbi.nlm.nih.gov/entrez/query.fcgi?cmd=Retrieve&db=Nucleotide&list_uids=56806513&dopt=GenBank&RID=W9GMSDH601R&log$=nuclalign&blast_rank=1) | 217 |
| RP14 | pepper | Receptor kinase [*Arabidopsis thaliana*] [gb|AAD02501.1|](http://www.ncbi.nlm.nih.gov/entrez/query.fcgi?cmd=Retrieve&db=Protein&list_uids=4105699&dopt=GenPept&RID=W81R31HH011&log$=prottop&blast_rank=16) | *Solanum lycopersicum* Tomato chromosome 2, C02HBa0320M09, complete sequence [gb|AC215438.1|](http://www.ncbi.nlm.nih.gov/entrez/query.fcgi?cmd=Retrieve&db=Nucleotide&list_uids=161789425&dopt=GenBank&RID=W9GMSDH601R&log$=nuclalign&blast_rank=1) | 209 |
| RP16 | pepper | NADH dehydrogenase subunit II [*Solanum tuberosum*] [emb|CAA63783.1|](http://www.ncbi.nlm.nih.gov/entrez/query.fcgi?cmd=Retrieve&db=Protein&list_uids=3334858&dopt=GenPept&RID=W81R31HH011&log$=protalign&blast_rank=3) | *Solanum tuberosum* mitochondrial trnC, trnN1, trnY, nad2 genes [emb|X93575.1|](http://www.ncbi.nlm.nih.gov/entrez/query.fcgi?cmd=Retrieve&db=Nucleotide&list_uids=3334857&dopt=GenBank&RID=W9GMSDH601R&log$=nuclalign&blast_rank=2) | 185 |
| RP18 | pepper | Cytochrome b6/f complex subunit IV [*Lactuca sativa*] [ref|YP_398359.2|](http://www.ncbi.nlm.nih.gov/entrez/query.fcgi?cmd=Retrieve&db=Protein&list_uids=127706198&dopt=GenPept&RID=W81R31HH011&log$=protalign&blast_rank=5) | *Solanum lycopersicum* complete chloroplast genome, cultivar IPA-6 [emb|AM087200.3|](http://www.ncbi.nlm.nih.gov/entrez/query.fcgi?cmd=Retrieve&db=Nucleotide&list_uids=113531108&dopt=GenBank&RID=W9GMSDH601R&log$=nuclalign&blast_rank=6) | 195 |
| RP19 | pepper | Unnamed protein product [*Vitis vinifera*] [emb|CAO38786.1|](http://www.ncbi.nlm.nih.gov/entrez/query.fcgi?cmd=Retrieve&db=Protein&list_uids=157349648&dopt=GenPept&RID=W81R31HH011&log$=prottop&blast_rank=1) |  | 392 |
| RP20 | pepper | Transport protein, putative [*Arabidopsis thaliana*] [ref|NP_568626.1|](http://www.ncbi.nlm.nih.gov/entrez/query.fcgi?cmd=Retrieve&db=Protein&list_uids=18422356&dopt=GenPept&RID=W81R31HH011&log$=protalign&blast_rank=3) | *Arabidopsis thaliana* transport protein, putative (AT5G43670) mRNA, complete cds [ref|NM_123733.1|](http://www.ncbi.nlm.nih.gov/entrez/query.fcgi?cmd=Retrieve&db=Nucleotide&list_uids=18422355&dopt=GenBank&RID=W9GMSDH601R&log$=nuclalign&blast_rank=1) | 186 |
| RP23 | pepper |  | *Medicago truncatula* clone mth2-11d4, complete sequence [gb|AC142394.19|](http://www.ncbi.nlm.nih.gov/entrez/query.fcgi?cmd=Retrieve&db=Nucleotide&list_uids=61806846&dopt=GenBank&RID=W9FBUUSE012&log$=nuclalign&blast_rank=2) | 210 |
| ST1 | tomato |  | *Solanum tuberosum* ribosomal protein S3 (Yrps3) gene, partial cds; mitochondrial genes encoding mitochondrial proteins [gb|AF096321.1|](http://www.ncbi.nlm.nih.gov/entrez/query.fcgi?cmd=Retrieve&db=Nucleotide&list_uids=4106848&dopt=GenBank&RID=W9HN7VMD01R&log$=nuclalign&blast_rank=1) | 435 |
| ST2 | tomato | S haplotype-specific F-box protein, putative [*Solanum demissum*] [gb|ABI34316.1|](http://www.ncbi.nlm.nih.gov/entrez/query.fcgi?cmd=Retrieve&db=Protein&list_uids=113205261&dopt=GenPept&RID=W83F9351013&log$=protalign&blast_rank=1) | *Solanum lycopersicum* cv. Heinz 1706, chromosome 5 BAC clone C05HBa0145P19, complete sequence [gb|AC209589.1|](http://www.ncbi.nlm.nih.gov/entrez/query.fcgi?cmd=Retrieve&db=Nucleotide&list_uids=157098801&dopt=GenBank&RID=W9HN7VMD01R&log$=nuclalign&blast_rank=1) | 455 |
| ST3 | tomato | Photosystem II CP47 chlorophyll apoprotein [*Terminalia catappa*] [gb|ABV65673.1|](http://www.ncbi.nlm.nih.gov/entrez/query.fcgi?cmd=Retrieve&db=Protein&list_uids=113205261&dopt=GenPept&RID=W83F9351013&log$=protalign&blast_rank=1) | *Solanum lycopersicum* complete chloroplast genome, cultivar IPA-6 [emb|AM087200.3|](http://www.ncbi.nlm.nih.gov/entrez/query.fcgi?cmd=Retrieve&db=Nucleotide&list_uids=113531108&dopt=GenBank&RID=326E8J10015&log$=nuclalign&blast_rank=1) | 309 |
| ST4 | tomato | Putative senescence-associated protein [*Pisum sativum*][dbj|BAB33421.1|](http://www.ncbi.nlm.nih.gov/entrez/query.fcgi?cmd=Retrieve&db=Protein&list_uids=13359451&dopt=GenPept&RID=326BVWHM013&log$=protalign&blast_rank=1) | *Solanum lycopersicum* 25S ribosomal RNA gene, complete sequence [gb|EU161982.1|](http://www.ncbi.nlm.nih.gov/entrez/query.fcgi?cmd=Retrieve&db=Nucleotide&list_uids=157863687&dopt=GenBank&RID=326E8J10015&log$=nuclalign&blast_rank=2) | 257 |
| ST5 | tomato | Kelch repeat-containing F-box family protein [*Arabidopsis thaliana*] [ref|NP_565238.1|](http://www.ncbi.nlm.nih.gov/entrez/query.fcgi?cmd=Retrieve&db=Protein&list_uids=18412854&dopt=GenPept&RID=326BVWHM013&log$=protalign&blast_rank=5) | *Vitis vinifera* contig VV78X234265.45, whole genome shotgun sequence [emb|AM425089.2|](http://www.ncbi.nlm.nih.gov/entrez/query.fcgi?cmd=Retrieve&db=Nucleotide&list_uids=147817702&dopt=GenBank&RID=326E8J10015&log$=nuclalign&blast_rank=1) | 251 |
| ST6 | tomato | BEL12_AG transposon polyprotein [*Anopheles gambiae*] [emb|CAJ14165.1|](http://www.ncbi.nlm.nih.gov/entrez/query.fcgi?cmd=Retrieve&db=Protein&list_uids=68697272&dopt=GenPept&RID=W83F9351013&log$=protalign&blast_rank=2) |  | 283 |
| ST7 | tomato | Unnamed protein product [*Vitis vinifera*] [emb|CAO64432.1|](http://www.ncbi.nlm.nih.gov/entrez/query.fcgi?cmd=Retrieve&db=Protein&list_uids=157342319&dopt=GenPept&RID=W83F9351013&log$=prottop&blast_rank=1) | *Solanum lycopersicum* chromosome 3 clone C03HBa0233O20, complete sequence [gb|EU124739.1|](http://www.ncbi.nlm.nih.gov/entrez/query.fcgi?cmd=Retrieve&db=Nucleotide&list_uids=157649047&dopt=GenBank&RID=W9HN7VMD01R&log$=nuclalign&blast_rank=1) | 355 |
| ST8 | tomato |  | *Solanum lycopersicum* clone BAC C09HBa0142I14, complete sequence [gb|EU180573.1|](http://www.ncbi.nlm.nih.gov/entrez/query.fcgi?cmd=Retrieve&db=Nucleotide&list_uids=157838030&dopt=GenBank&RID=W9JCREXZ016&log$=nuclalign&blast_rank=1) | 305 |
| ST9 | tomato | Ribosomal protein L16 [*Solanum lycopersicum*] [ref|YP_514889.1|](http://www.ncbi.nlm.nih.gov/entrez/query.fcgi?cmd=Retrieve&db=Protein&list_uids=89280672&dopt=GenPept&RID=326BVWHM013&log$=protalign&blast_rank=4) | *Solanum bulbocastanum* cultivar PT29 chloroplast, complete genome [gb|DQ347958.1|](http://www.ncbi.nlm.nih.gov/entrez/query.fcgi?cmd=Retrieve&db=Nucleotide&list_uids=84371874&dopt=GenBank&RID=326E8J10015&log$=nuclalign&blast_rank=1) | 373 |
| ST10 | tomato |  | *S.lycopersicum* DNA sequence from clone LE_HBa-190C13 on chromosome 4, complete sequence [emb|CU210847.3|](http://www.ncbi.nlm.nih.gov/entrez/query.fcgi?cmd=Retrieve&db=Nucleotide&list_uids=124107491&dopt=GenBank&RID=W9JCREXZ016&log$=nuclalign&blast_rank=1) | 159 |
| ST11 | tomato |  | *L.esculentum* Le-hsf8 gene for heat stress transcription factor 8 [emb|X67599.1|LEHSF8](http://www.ncbi.nlm.nih.gov/entrez/query.fcgi?cmd=Retrieve&db=Nucleotide&list_uids=19259&dopt=GenBank&RID=W9K6RYSC01R&log$=nuclalign&blast_rank=1) | 212 |
| ST12 | tomato | Putative retrotransposon protein, identical [*Solanum demissum*] [gb|AAT38724.1|](http://www.ncbi.nlm.nih.gov/entrez/query.fcgi?cmd=Retrieve&db=Protein&list_uids=47824950&dopt=GenPept&RID=W83F9351013&log$=protalign&blast_rank=1) | *Solanum lycopersicum* DNA, chromosome 8, clone: C08HBa0067I16, complete sequence [dbj|AP009518.1|](http://www.ncbi.nlm.nih.gov/entrez/query.fcgi?cmd=Retrieve&db=Nucleotide&list_uids=158258995&dopt=GenBank&RID=W9HN7VMD01R&log$=nuclalign&blast_rank=1) | 305 |
| ST13 | tomato | ABC transporter, ATP-binding protein [*Vibrionales bacterium* SWAT-3] [ref|ZP_01812900.1|](http://www.ncbi.nlm.nih.gov/entrez/query.fcgi?cmd=Retrieve&db=Protein&list_uids=148976157&dopt=GenPept&RID=W86CWTMC015&log$=protalign&blast_rank=1) |  | 210 |
| ST14 | tomato | RNase H family protein [*Solanum demissum*] [gb|ABI34372.1|](http://www.ncbi.nlm.nih.gov/entrez/query.fcgi?cmd=Retrieve&db=Protein&list_uids=113205385&dopt=GenPept&RID=W83F9351013&log$=protalign&blast_rank=4) | *Solanum lycopersicum* genomic DNA, chromosome 8, complete sequence, clone: C08SLe0028K16 [dbj|AP009395.1|](http://www.ncbi.nlm.nih.gov/entrez/query.fcgi?cmd=Retrieve&db=Nucleotide&list_uids=146424715&dopt=GenBank&RID=W9HN7VMD01R&log$=nuclalign&blast_rank=1) | 323 |
| ST15 | tomato | PetD [*Hordeum vulgare* subsp. vulgare] [emb|CAA32268.1|](http://www.ncbi.nlm.nih.gov/entrez/query.fcgi?cmd=Retrieve&db=Protein&list_uids=11598&dopt=GenPept&RID=W83F9351013&log$=protalign&blast_rank=4) | *Solanum lycopersicum* complete chloroplast genome, cultivar IPA-6 [emb|AM087200.3|](http://www.ncbi.nlm.nih.gov/entrez/query.fcgi?cmd=Retrieve&db=Nucleotide&list_uids=113531108&dopt=GenBank&RID=W9HN7VMD01R&log$=nuclalign&blast_rank=1) | 193 |
| ST19 | tomato | Similar to u1 small nuclear ribonucleoprotein C [*Solanum lycopersicum*] [dbj|BAD95791.1|](http://www.ncbi.nlm.nih.gov/entrez/query.fcgi?cmd=Retrieve&db=Protein&list_uids=62751087&dopt=GenPept&RID=W86CWTMC015&log$=protalign&blast_rank=1) | *Solanum lycopersicum* mRNA for similar to u1 small nuclear ribonucleoprotein C, complete cds, clone:FC01AC10 [dbj|AB211519.1|](http://www.ncbi.nlm.nih.gov/entrez/query.fcgi?cmd=Retrieve&db=Nucleotide&list_uids=62751086&dopt=GenBank&RID=W9K6RYSC01R&log$=nuclalign&blast_rank=1) | 401 |
| ST26 | tomato | Tyrosine specific protein phosphatase family protein [*Capsicum annuum*] [gb|AAX20039.1|](http://www.ncbi.nlm.nih.gov/entrez/query.fcgi?cmd=Retrieve&db=Protein&list_uids=60459387&dopt=GenPept&RID=W86CWTMC015&log$=protalign&blast_rank=4) | *Solanum lycopersicum* cDNA, clone: FC22BF07, HTC in fruit [dbj|AK246745.1|](http://www.ncbi.nlm.nih.gov/entrez/query.fcgi?cmd=Retrieve&db=Nucleotide&list_uids=148537979&dopt=GenBank&RID=W9K6RYSC01R&log$=nuclalign&blast_rank=1) | 377 |
| ST27 | tomato | Sgt1b [*Capsicum annuum*] [gb|AAX83943.1|](http://www.ncbi.nlm.nih.gov/entrez/query.fcgi?cmd=Retrieve&db=Protein&list_uids=62467587&dopt=GenPept&RID=W7926W67015&log$=prottop&blast_rank=1) | *Solanum tuberosum* SGT1 gene, complete cds [gb|AY615272.1|](http://www.ncbi.nlm.nih.gov/entrez/query.fcgi?cmd=Retrieve&db=Nucleotide&list_uids=51511449&dopt=GenBank&RID=W9DBDT7F016&log$=nuclalign&blast_rank=2) | 191 |
| ST28 | tomato | Hypothetical protein [ref|XP_001524063.1|](http://www.ncbi.nlm.nih.gov/entrez/query.fcgi?cmd=Retrieve&db=Protein&list_uids=149236371&dopt=GenPept&RID=W7926W67015&log$=prottop&blast_rank=1) | *Solanum lycopersicum* Tomato chromosome 2, C02SLe0034H10, complete sequence [gb|AC215453.1|](http://www.ncbi.nlm.nih.gov/entrez/query.fcgi?cmd=Retrieve&db=Nucleotide&list_uids=161789440&dopt=GenBank&RID=W9DBDT7F016&log$=nuclalign&blast_rank=1) | 228 |
| ST30 | tomato | ORF107c [*Pinus koraiensis*] [ref|YP_001152220.1|](http://www.ncbi.nlm.nih.gov/entrez/query.fcgi?cmd=Retrieve&db=Protein&list_uids=145408596&dopt=GenPept&RID=W7926W67015&log$=prottop&blast_rank=4) | *Delphinium elatum* chloroplast 23S - 5S ribosomal RNA genes, internal transcribed spacer [gb|L29445.1|DELCP](http://www.ncbi.nlm.nih.gov/entrez/query.fcgi?cmd=Retrieve&db=Nucleotide&list_uids=1212723&dopt=GenBank&RID=W9DBDT7F016&log$=nuclalign&blast_rank=4) | 159 |
| ST31 | tomato | Ribosomal protein S14 [*Solanum lycopersicum*] [ref|YP_514850.1|](http://www.ncbi.nlm.nih.gov/entrez/query.fcgi?cmd=Retrieve&db=Protein&list_uids=89280633&dopt=GenPept&RID=W7926W67015&log$=prottop&blast_rank=1) | *Solanum lycopersicum* complete chloroplast genome, cultivar IPA-6 [emb|AM087200.3|](http://www.ncbi.nlm.nih.gov/entrez/query.fcgi?cmd=Retrieve&db=Nucleotide&list_uids=113531108&dopt=GenBank&RID=W9DBDT7F016&log$=nuclalign&blast_rank=1) | 662 |
| ST32 | tomato | Unnamed protein product [*Vitis vinifera*] [emb|CAO45037.1|](http://www.ncbi.nlm.nih.gov/entrez/query.fcgi?cmd=Retrieve&db=Protein&list_uids=157353127&dopt=GenPept&RID=W7926W67015&log$=prottop&blast_rank=1) | *Vitis vinifera* contig VV78X033551.38, whole genome shotgun sequence [emb|AM483114.2|](http://www.ncbi.nlm.nih.gov/entrez/query.fcgi?cmd=Retrieve&db=Nucleotide&list_uids=147783179&dopt=GenBank&RID=W9DBDT7F016&log$=nuclalign&blast_rank=1) | 250 |
| ST33 | tomato |  | *Solanum lycopersicum* Tomato chromosome 2, C02HBa0122C10, complete sequence [gb|AC215387.1|](http://www.ncbi.nlm.nih.gov/entrez/query.fcgi?cmd=Retrieve&db=Nucleotide&list_uids=161789374&dopt=GenBank&RID=W9DBDT7F016&log$=nuclalign&blast_rank=1) | 186 |
| ST34 | tomato | Unnamed protein product [*Vitis vinifera*] [emb|CAO63423.1|](http://www.ncbi.nlm.nih.gov/entrez/query.fcgi?cmd=Retrieve&db=Protein&list_uids=157341967&dopt=GenPept&RID=W7926W67015&log$=prottop&blast_rank=1) | *Arabidopsis thaliana* PLL4 (POLTERGEIST LIKE 4); protein phosphatase type 2C (PLL4) mRNA, complete cds [ref|NM_128448.3|](http://www.ncbi.nlm.nih.gov/entrez/query.fcgi?cmd=Retrieve&db=Nucleotide&list_uids=30684051&dopt=GenBank&RID=W9DBDT7F016&log$=nuclalign&blast_rank=1) | 247 |
| ST35 | tomato | Unnamed protein product [*Vitis vinifera*] [emb|CAO45037.1|](http://www.ncbi.nlm.nih.gov/entrez/query.fcgi?cmd=Retrieve&db=Protein&list_uids=157353127&dopt=GenPept&RID=W7926W67015&log$=prottop&blast_rank=1) | *Vitis vinifera* contig VV78X234265.45, whole genome shotgun sequence [emb|AM425089.2|](http://www.ncbi.nlm.nih.gov/entrez/query.fcgi?cmd=Retrieve&db=Nucleotide&list_uids=147817702&dopt=GenBank&RID=W9DBDT7F016&log$=nuclalign&blast_rank=1) | 250 |
| ST36 | tomato | Ribosomal protein S14 [*Solanum lycopersicum*] [ref|YP_514850.1|](http://www.ncbi.nlm.nih.gov/entrez/query.fcgi?cmd=Retrieve&db=Protein&list_uids=89280633&dopt=GenPept&RID=W7926W67015&log$=prottop&blast_rank=1) | *Solanum tuberosum* cultivar Desiree chloroplast, complete genome [gb|DQ386163.1|](http://www.ncbi.nlm.nih.gov/entrez/query.fcgi?cmd=Retrieve&db=Nucleotide&list_uids=88656783&dopt=GenBank&RID=W9DBDT7F016&log$=nuclalign&blast_rank=2) | 236 |
| ST37 | tomato | Chlorophyll A-B binding family protein [*Arabidopsis thaliana*] [ref|NP_177783.1|](http://www.ncbi.nlm.nih.gov/entrez/query.fcgi?cmd=Retrieve&db=Protein&list_uids=15223108&dopt=GenPept&RID=W7926W67015&log$=protalign&blast_rank=2) | *S.lycopersicum* DNA sequence from clone SL_MboI-59M16 on chromosome 4, complete sequence [emb|CU179634.6|](http://www.ncbi.nlm.nih.gov/entrez/query.fcgi?cmd=Retrieve&db=Nucleotide&list_uids=124053137&dopt=GenBank&RID=W9DBDT7F016&log$=nuclalign&blast_rank=1) | 297 |
| SE1 | Eggplant | Hypothetical protein SpolCp017 [S*pinacia oleracea*] ref|NP_054927.1| |  | 280 |
| SE2 | eggplant | Putative retrotransposon protein, identical [*Solanum demissum*] [gb|AAT38724.1|](http://www.ncbi.nlm.nih.gov/entrez/query.fcgi?cmd=Retrieve&db=Protein&list_uids=47824950&dopt=GenPept&RID=W83F9351013&log$=protalign&blast_rank=2) | *S.lycopersicum* DNA sequence from clone LE_HBa-311A10 on chromosome 4, complete sequence [emb|CU222537.4|](http://www.ncbi.nlm.nih.gov/entrez/query.fcgi?cmd=Retrieve&db=Nucleotide&list_uids=123711044&dopt=GenBank&RID=W9HN7VMD01R&log$=nuclalign&blast_rank=1) | 276 |
| SE3 | Eggplant | Cation-chloride co-transporter [*Nicotiana tabacum*] gb|AAC49874.1| |  | 258 |
| SE4 | eggplant |  | *S.lycopersicum* DNA sequence from clone SL_MboI-33N19 on chromosome 4, complete sequence [emb|CU234210.4|](http://www.ncbi.nlm.nih.gov/entrez/query.fcgi?cmd=Retrieve&db=Nucleotide&list_uids=124221865&dopt=GenBank&RID=W9HN7VMD01R&log$=nuclalign&blast_rank=1) | 254 |
| SE5 | eggplant |  | *Vitis vinifera*, whole genome shotgun sequence, contig VV78X178746.5, clone ENTAV 115 [emb|AM486247.1|](http://www.ncbi.nlm.nih.gov/entrez/query.fcgi?cmd=Retrieve&db=Nucleotide&list_uids=123699157&dopt=GenBank&RID=W9JCREXZ016&log$=nuclalign&blast_rank=1) | 112 |
| SE6 | eggplant | Similar to TRAF interacting protein [*Monodelphis domestica*] [ref|XP_001378213.1|](http://www.ncbi.nlm.nih.gov/entrez/query.fcgi?cmd=Retrieve&db=Protein&list_uids=126336044&dopt=GenPept&RID=W83F9351013&log$=protalign&blast_rank=1) |  | 393 |
| SE7 | eggplant | Orf315 [*Beta vulgaris subsp. vulgaris*] [ref|NP_063979.1|](http://www.ncbi.nlm.nih.gov/entrez/query.fcgi?cmd=Retrieve&db=Protein&list_uids=9838367&dopt=GenPept&RID=W83F9351013&log$=protalign&blast_rank=1) |  | 213 |
| SE8 | eggplant | Cytochrome b6/f complex subunit 4 [*Lactuca sativa*] [dbj|BAE47626.1|](http://www.ncbi.nlm.nih.gov/entrez/query.fcgi?cmd=Retrieve&db=Protein&list_uids=78675200&dopt=GenPept&RID=W83F9351013&log$=protalign&blast_rank=3) | *Solanum lycopersicum* complete chloroplast genome, cultivar IPA-6 [emb|AM087200.3|](http://www.ncbi.nlm.nih.gov/entrez/query.fcgi?cmd=Retrieve&db=Nucleotide&list_uids=113531108&dopt=GenBank&RID=W9HN7VMD01R&log$=nuclalign&blast_rank=1) | 194 |
| SE9 | eggplant | NADH dehydrogenase subunit 3 [*Solanum lycopersicum*] [ref|YP_514857.1|](http://www.ncbi.nlm.nih.gov/entrez/query.fcgi?cmd=Retrieve&db=Protein&list_uids=89280640&dopt=GenPept&RID=W85AUU80013&log$=protalign&blast_rank=1) | *Solanum lycopersicum* complete chloroplast genome, cultivar IPA-6 [emb|AM087200.3|](http://www.ncbi.nlm.nih.gov/entrez/query.fcgi?cmd=Retrieve&db=Nucleotide&list_uids=113531108&dopt=GenBank&RID=W9JCREXZ016&log$=nuclalign&blast_rank=1) | 483 |
| SE11 | eggplant | GNS1/SUR4 membrane family protein [*Arabidopsis thaliana*] [ref|NP_195401.1|](http://www.ncbi.nlm.nih.gov/entrez/query.fcgi?cmd=Retrieve&db=Protein&list_uids=15234538&dopt=GenPept&RID=W85AUU80013&log$=protalign&blast_rank=2) | *Arabidopsis thaliana* GNS1/SUR4 membrane family protein (AT4G36830) mRNA, complete cds [ref|NM_119847.3|](http://www.ncbi.nlm.nih.gov/entrez/query.fcgi?cmd=Retrieve&db=Nucleotide&list_uids=145354494&dopt=GenBank&RID=W9JCREXZ016&log$=nuclalign&blast_rank=3) | 309 |
| SE13 | eggplant | Ribosomal protein S14 [*Solanum lycopersicum*] [ref|YP_514850.1|](http://www.ncbi.nlm.nih.gov/entrez/query.fcgi?cmd=Retrieve&db=Protein&list_uids=89280633&dopt=GenPept&RID=W85AUU80013&log$=protalign&blast_rank=1) | *Lycopersicon esculentum* cultivar LA3023 chloroplast, complete [gb|DQ347959.1|](http://www.ncbi.nlm.nih.gov/entrez/query.fcgi?cmd=Retrieve&db=Nucleotide&list_uids=84371962&dopt=GenBank&RID=W9JCREXZ016&log$=nuclalign&blast_rank=3) | 201 |
| SE14 | eggplant | Retrotransposon protein [*Beta vulgaris*] [gb|ABM55240.1|](http://www.ncbi.nlm.nih.gov/entrez/query.fcgi?cmd=Retrieve&db=Protein&list_uids=121501699&dopt=GenPept&RID=W85AUU80013&log$=protalign&blast_rank=3) | *Solanum lycopersicum* chromosome 10 clone C10HBa0115K16, complete [gb|AC193780.1|](http://www.ncbi.nlm.nih.gov/entrez/query.fcgi?cmd=Retrieve&db=Nucleotide&list_uids=118344476&dopt=GenBank&RID=W9JCREXZ016&log$=nuclalign&blast_rank=1) | 262 |
| SE16 | eggplant | Hypothetical protein NitaMp023 [*Nicotiana tabacum*] [ref|YP_173370.1|](http://www.ncbi.nlm.nih.gov/entrez/query.fcgi?cmd=Retrieve&db=Protein&list_uids=57013896&dopt=GenPept&RID=W85AUU80013&log$=protalign&blast_rank=1) | Nicotiana tabacum mitochondrial DNA, complete genome [dbj|BA000042.1|](http://www.ncbi.nlm.nih.gov/entrez/query.fcgi?cmd=Retrieve&db=Nucleotide&list_uids=56806513&dopt=GenBank&RID=W9JCREXZ016&log$=nuclalign&blast_rank=1) | 365 |
| SE18 | eggplant | Generic methyltransferase [*Medicago truncatula*] [gb|ABN08826.1|](http://www.ncbi.nlm.nih.gov/entrez/query.fcgi?cmd=Retrieve&db=Protein&list_uids=124360854&dopt=GenPept&RID=W86CWTMC015&log$=prottop&blast_rank=3) | *Medicago truncatula* clone mth2-116m7, complete sequence [gb|AC149494.4|](http://www.ncbi.nlm.nih.gov/entrez/query.fcgi?cmd=Retrieve&db=Nucleotide&list_uids=50355772&dopt=GenBank&RID=W9K6RYSC01R&log$=nuclalign&blast_rank=1) | 333 |
| SE19 | eggplant | At1g75460/F1B16_22 [*Arabidopsis thaliana*] [gb|AAL77655.1|](http://www.ncbi.nlm.nih.gov/entrez/query.fcgi?cmd=Retrieve&db=Protein&list_uids=18700087&dopt=GenPept&RID=W86CWTMC015&log$=prottop&blast_rank=2) | *Arabidopsis thaliana* ATP-dependent protease La (LON) domain-containing protein (AT1G19740) mRNA, complete cds [ref|NM_101830.2|](http://www.ncbi.nlm.nih.gov/entrez/query.fcgi?cmd=Retrieve&db=Nucleotide&list_uids=30686518&dopt=GenBank&RID=W9K6RYSC01R&log$=nuclalign&blast_rank=3) | 559 |
| SE21 | eggplant |  | *Lycopersicon chmielewskii* putative copper/zinc superoxide dismutase (CT148) gene, CT148-7206b allele, partial cds [gb|DQ103425.1|](http://www.ncbi.nlm.nih.gov/entrez/query.fcgi?cmd=Retrieve&db=Nucleotide&list_uids=73808603&dopt=GenBank&RID=W9K6RYSC01R&log$=nuclalign&blast_rank=1) | 308 |
