# Supplementary material for: Inter-Species Grafting Caused Extensive and Heritable Alterations of DNA Methylation in Solanaceae Plants
Source: PLoS One. 2013 Apr 16;8(4):e61995. doi: 10.1371/journal.pone.0061995 (PMC3628911; doi:10.1371/journal.pone.0061995)
Supplement: Table S5 — Chi-squared test for statistical significance in frequencies of methylated cytosines vs. total cytosines (based on BS-seq) in each of the three sequence contexts, CG, CHG and CHH, as well as total C for each of the three analyzed sequences, between the seed-plant control and each of the self- and hetero-grafted plants by using R package. (DOC) [file pone.0061995.s006.doc]

| Grafted plant | Scion | Rootstock | Sample DNA analyzed | S1 plants used for analysis* |
| --- | --- | --- | --- | --- |
| eT1 | Tomato | Eggplant | Tomato | 10 |
| eT2 | Tomato | Eggplant | Tomato | 8 |
| eT3 | Tomato | Eggplant | Tomato | -- |
| tE1 | Eggplant | Tomato | Eggplant | 1 |
| tE2 | Eggplant | Tomato | Eggplant | 1 |
| tE3 | Eggplant | Tomato | Eggplant | -- |
| Pt1 | Tomato | Pepper | Pepper | -- |
| Pt2 | Tomato | Pepper | Pepper | -- |
| Pt3 | Tomato | Pepper | Pepper | -- |

**Table S5.** Details of the grafted plants used in the MSAP analysis.

*S1 plants: selfed plants of generation one.
