# Supplementary material for: Inter-Species Grafting Caused Extensive and Heritable Alterations of DNA Methylation in Solanaceae Plants
Source: PLoS One. 2013 Apr 16;8(4):e61995. doi: 10.1371/journal.pone.0061995 (PMC3628911; doi:10.1371/journal.pone.0061995)
Supplement: Table S6 — Details of the grafted plants used in this study. (DOC) [file pone.0061995.s007.doc]

**Table S5.** Chi-squared test for statistical significance in frequencies of methylated cytosines vs. total cytosines (based on BS-seq) in each of the three sequence contexts, CG, CHG and CHH, as well as total for each of the three analyzed sequences, between the seed-plant control and each of the self- and hetero-grafted plants by using R package.

|  |  | tT | eT1 | eT2 | S1 of eT1 | S1 of eT2 |
| --- | --- | --- | --- | --- | --- | --- |
| ST4 | ^m^CG | 0.041* | 0.001** | 5.78E-08** | 3.71E-04** | 0.046* |
|  | ^m^CHG | 0.057 | 0.001** | 8.59E-05** | 0.076 | 0.059 |
|  | ^m^CHH | 0.005** | 1.48E-08** | 7.95E-07** | 0.752 | 3.75E-06** |
|  | total ^m^C | 0.004** | 4.62E-08** | 5.63E-09** | 0.033* | 9.57E-05** |
|  |  | eE | tE1 | tE2 | S1 of tE1 | S1 of tE2 |
| SE1 | ^m^CG | 0.933 | 4.94E-10** | 9.38E-19** | 2.13E-17** | 7.19E-21** |
|  | ^m^CHG | 0.288 | 4.57E-05** | 7.06E-11** | 1.26E-09** | 3.37E-11** |
|  | ^m^CHH | 0.316 | 0.001** | 2.40E-05** | 3.86E-04** | 2.40E-05** |
|  | total ^m^C | 0.823 | 8.92E-16** | 7.60E-30** | 2.53E-26** | 8.86E-32** |
|  |  | eE | tE1 | tE2 | S1 of tE1 | S1 of tE2 |
| SE2 | ^m^CG | 0.058 | 0.343 | 0.040* | 0.764 | 0.058 |
|  | ^m^CHG | NA | NA | 0.013* | 0.005** | 0.003** |
|  | ^m^CHH | 0.009** | 0.162 | 0.165 | 0.560 | 0.024* |
|  | total ^m^C | 0.018* | 0.173 | 0.109 | 0.230 | 0.007** |

NA (not applicable) meaning that there was no ^m^CHG site found by bisulfite sequencing and hence Chi-squared test cannot performed.

* and ** represent significant difference at the 0.05 and ≤ 0.01 statistical levels, respectively.
